# Enhancement of Apiaceae pre-germination embryo growth, mericarp ageing resilience and germination differs between hormone, gas plasma, and hydropriming technologies

**DOI:** 10.1007/s00425-025-04900-0

**Published:** 2026-01-03

**Authors:** Lena M. M. Fatelnig, Matthew Walker, Giles Grainge, James E. Hourston, Sue Kennedy, Veronika Turečková, Ondřej Novák, Danuše Tarkowská, Miroslav Strnad, Kazumi Nakabayashi, Tina Steinbrecher, Gerhard Leubner-Metzger

**Affiliations:** 1 Department of Biological Sciences, Seed Biology and Technology Group, Royal Holloway University of London, Egham, TW20 0EX UK; 2 Tozer Seeds Ltd, Cobham, KT11 3EH UK; 3 Syngenta Ltd., Jealott’s Hill International Research Centre, Bracknell, RG42 6EY UK; 4 Eden Research plc, Milton Park, Oxforshire, OX14 4SA UK; 5 Elsoms Seeds Ltd, Spalding, Lincolnshire, PE11 1QG UK; 6 Laboratory of Growth Regulators, Institute of Experimental Botany, Palacký University Olomouc, Czech Academy of Sciences and Faculty of Science, 77900 Olomouc, Czech Republic; 7 Department of Agro-environmental Science, Obihiro University of Agriculture and Veterinary Medicine, Obihiro, Hokkaido 080-8555 Japan

**Keywords:** *Daucus carota* (carrot), *Pastinaca sativa* (parsnip), Morphological seed dormancy, Gas plasma seed priming, Hormone seed hydropriming, Pre-germination embryo growth

## Abstract

**Main conclusion:**

Enhanced Apiaceae germination performance by seed priming involves promoting pre-germination growth of the underdeveloped (small) embryos, reduction in hormone contents, and priming with abscisic acid (ABA) improved ageing resilience.

**Abstract:**

Different seed priming technologies are used to improve germination performance and seedling vigour of vegetable crops. *Daucus carota* (carrot), *Pastinaca sativa* (parsnip), and other Apiaceae produce morphologically dormant single-seeded fruit halves (mericarps) as dispersal units. In mature mericarps, the underdeveloped (small) embryo is embedded in abundant endosperm tissue, and pre-germination embryo growth to a critical embryo:seed (E:S) length ratio is a requirement for the completion of germination by radicle emergence. We investigated how hydropriming and additive priming with gibberellins (GA), abscisic acid (ABA), and gas plasma-activated water (GPAW) affected carrot and parsnip mericarp germination and ageing sensitivity accessed using a wet ageing assay (80% RH, 42 °C). Carrot and parsnip mericarp priming enhanced germination speed (germination rate GR_50%_), maximal germination percentage (*G*_max_), and germination vigour. This was associated with enhanced pre-emergence embryo growth inside hydroprimed, hormone-primed, and GPAW-primed mericarps. Hydropriming affected the hormone contents and ABA sensitivity of parsnip mericarps. It reduced the contents of bioactive GAs and indole-3-acetic acid ~ 2.1 and ~ 7.7-fold, and of the germination inhibitors ABA and *cis*-(+)-12-oxo-phytodienoic acid ~ 9.2 and ~ 6.0-fold, respectively. Hydroprimed carrot and parsnip mericarps were more sensitive in the wet ageing assay. GPAW-priming increased carrot salinity tolerance but did not increase its wet ageing resilience to a controlled deterioration treatment (CDT). In contrast, GPAW-priming increased the wet ageing resilience of many other vegetable seeds and cereal grains. ABA-priming not only enhanced embryo growth and germination performance, it also increased the wet ageing resilience of carrot and parsnip mericarps. We conclude that ABA-priming and GPAW-priming are promising technologies to improve vigour and wet ageing resilience of primed seeds.

**Supplementary Information:**

The online version contains supplementary material available at 10.1007/s00425-025-04900-0.

## Introduction

Climate change has intensified the frequency, severity, and duration of extreme weather events. Extreme heat, cold, and drought pose the greatest threats to global crop production (Lesk et al. 2022; Furtak and Wolinska 2023; Erfatpour et al. 2024). Seed germination and early seedling growth are one of the most vulnerable plant life cycle stages, with plant establishment at risk from abiotic (e.g. heat, cold, drought, salinity) and biotic (e.g. pathogenic microbes, weeds) environmental stresses (Finch-Savage and Leubner-Metzger 2006; Finch-Savage and Bassel 2016; Fernandez-Pascual et al. 2019; Walck et al. 2011; Steinbrecher et al. 2025b). High-quality crop seeds are therefore central to the sustainability and resilience of agri-food systems. Environmentally friendly innovative seed technologies include various priming, coating, pelleting, and other processing methods to aid sowing and/or enhance crop seed performance and stress resilience in the field (Ignatz et al. 2019; Bruggink 2022; Pedrini et al. 2020; Rawal et al. 2024; Pagano et al. 2023). A “one size fits all” strategy for applying these seed technologies to different species is impeded by the enormous diversity of their diaspores (dispersal units, here seeds or fruits) in morphological (e.g. embryo:seed size ratios, seed and fruit coat characteristics) and physiological (e.g. dormancy class, hormone and temperature sensitivities) properties (Carta et al. 2024; Willis et al. 2014; Baskin and Baskin 2014). Further to this, primed seeds of many species have increased ageing sensitivity and therefore a reduced storability (‘shelf-life’) due to faster deterioration processes (Pagano et al. 2023; Fabrissin et al. 2021; Bruggink et al. 1999; Chojnowski et al. 1997; Argerich et al. 1989; Zinsmeister et al. 2020).

Seed priming, a technique used commercially with many vegetables, flowers, and field crops, is a pre-sowing technique to physiologically enhance seed performance and seedling vigour. This involves controlled hydration (phases I and II of the water uptake curve) to allow metabolic activation, but without permitting the seed to proceed to water uptake phase III or visible radicle protrusion (Bruggink 2022; Corbineau et al. 2023; Weitbrecht et al. 2011; Pagano et al. 2023; Bradford 1986). The controlled hydration method (e.g. hydropriming, osmopriming) is followed by drying seeds back to a low moisture content similar to the original air-dry state. During seed hydropriming, which is often conducted using the drum priming technique, water uptake is restricted by adding a defined limited amount of water or over a defined short time period. During seed osmopriming, reduced water potential is generated by adding osmolytes (e.g. polyethylene glycol or salts) to restrict the transition from water uptake phase II to III. These priming treatments enhance germination in that the times of the seed population for 1% and 50%, *T*_1%_ and *T*_50%_, respectively, are reached earlier. The effect of seed priming, which comprises hydration plus drying, is therefore accelerated and synchronised germination leading to uniform and vigorous seedling establishment even upon stress (Corbineau et al. 2023; Fatelnig et al. 2024; Ibrahim 2016; Pagano et al. 2023). The mechanisms underpinning seed priming have mainly been studied with non-dormant (ND) and physiologically dormant (PD) species in which the embryo is fully developed and occupies most of the mature diaspore’s internal structure at dispersal (Corbineau et al. 2023; Gran et al. 2025; Pagano et al. 2023; Fatelnig et al. 2024; Yao et al. 2025b).

Far less is known about the mechanisms underpinning the priming of species with morphological (MD) and morphophysiological (MPD) dormancy. These MD/MPD diaspores are characterised by containing underdeveloped (small) embryos embedded in the abundant living endosperm tissue in their mature state at dispersal (Baskin and Baskin 2023; Zhang et al. 2019; Porceddu et al. 2017; Carta et al. 2024). Underdeveloped (small) embryos embedded in the abundant living endosperm tissue are a hallmark of the Apiaceae (carrot family), including the important vegetable crops carrot, celery, parsnip, fennel, and parsley, which are known for their germination problems in horticultural practice (Robinson 1954). The Apiaceae produce dry schizocarps as fruits, which split into individual mericarps upon maturity. These mericarps are the dispersal and germination units; they constitute dry single-seeded indehiscent fruit halves with a dead outer pericarp (fruit coat) encasing the seed (Liu et al. 2006; Wojewodzka et al. 2019; Walker et al. 2025; Nakabayashi et al. 2025). The underdeveloped embryos must first grow inside the imbibed MD/MPD mericarps to reach a critical embryo:seed (E:S) length ratio before germination can be completed by radicle emergence (Gray et al. 1990; Walker et al. 2021; Homrichhausen et al. 2003; Jacobsen and Pressman 1979; Jacobsen et al. 1976; Visscher et al. 2022; Baskin and Baskin 2014; Vandelook et al. 2012). The physiological component of this dormancy is usually lost in horticultural Apiaceae crops, including *Daucus carota* (carrot) (Homrichhausen et al. 2003; Nascimento et al. 2013; Nakabayashi et al. 2025) and *Apium graveolens* (celery) (Biddington and Thomas 1978; Walker et al. 2021, 2025; Van der Toorn and Karssen 1992; Van der Toorn 1990), and these species consequently have MD. Ecophysiological studies suggest that there is a very close association between MD and MPD with numerous species capable of exhibiting both dormancy classes, as well as multiple levels of MPD, within a single species (including *Pastinaca sativa*, parsnip), population, or plant (Baskin and Baskin 1979; Zardari et al. 2019; Hawkins et al. 2010).

We show here that priming of carrot and parsnip mericarps is associated with pre-germination growth of their underdeveloped embryos inside the imbibed mericarps, altered hormone contents and sensitivities, including for gibberellins (GA) and abscisic acid (ABA), and that additive priming with ABA or gas plasma-activated water (GPAW) can improve priming intensity and storage resilience. Gas plasma is often defined as “the fourth state of matter” due to its high energetic state, and non-thermal (cold, non-equilibrium) atmospheric plasma produced at ambient temperature and pressure has promising applications in sustainable food production (Bourke et al. 2018; Ito et al. 2018; Zhou et al. 2020). The unique aspects of ABA, GA, and GPAW priming on the pre-germination embryo growth and ageing sensitivities of Apiaceae vegetable mericarps are revealed and discussed in the context of developing innovative seed technologies to enhance crop germination performance and abiotic stress resilience in the field.

## Materials and methods

### Plant materials

Mericarps of *Daucus carota* L. (carrot) cultivars Nerac (lot no. E56490 (2018), graded 1.6–1.8 mm), Newcastle (lot no. E57968), and *Pastinaca sativa* L. (parsnip) F1 hybrid cultivars Panorama (#1 lot no. E64291 (2019), #3 lot no. E38814/E38817, and corresponding primed #3 lot no. RHTSB6P/RHTSB2W67) and Pacific (#1 lot no. E64292 (2019)) were provided by Elsoms Seeds Ltd. (Spalding, Lincolnshire, UK). Parsnip mericarp lots #1 and #3 were pre-washed, and all parsnip lots were in addition size-graded to reduce the effect of seed weight and maturity (remove small immature seeds from secondary and tertiary umbels) on germination characteristics (Hendrix 1984). Calibres obtained for parsnip ranged from 3.50 to 5.25 mm; of these, the 4.75–5.00 mm calibres were used for the experiments. For #3, the germination results at different temperatures for the 4.75–5.00 mm (E38814) and 3.75–4.00 mm (E38817) calibres did not differ (Nakabayashi et al. 2025). Mericarp aliquots for experimental use were stored at 4 °C in airtight containers containing silica.

### Germination assays

**Fig. 1:**
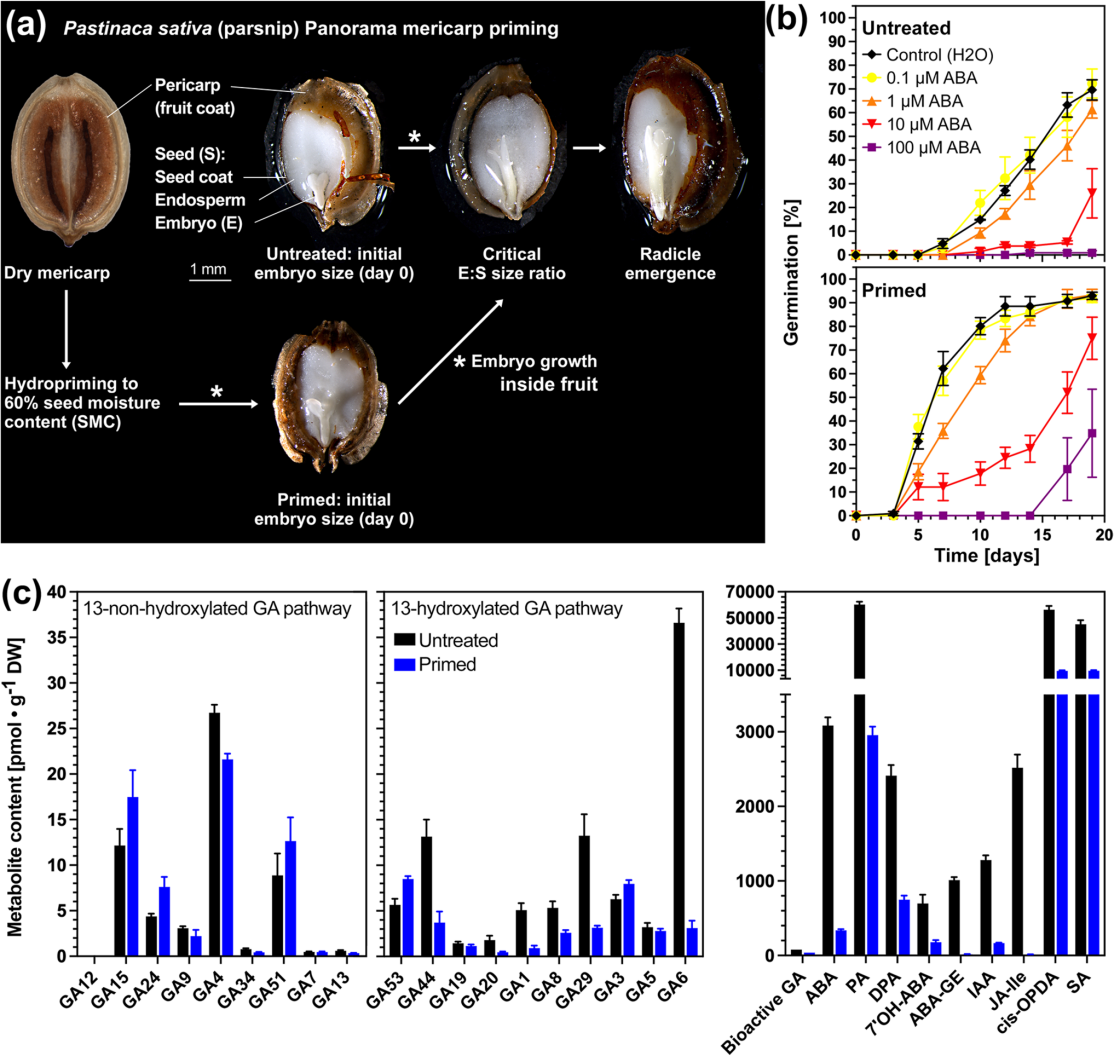
The effect of hydropriming on embryo growth, hormone contents, and sensitivity of parsnip mericarps. **a** Microscopic images of pre-germination embryo growth of morphologically dormant (MD) *Pastinaca sativa* cultivar Panorama mericarps during germination and hydropriming. **b** Germination kinetics of untreated and primed parsnip Panorama mericarps (lot #3) without and with abscisic acid (ABA) added during imbibition at 30 °C/20 °C day/night (8 h light/16 h darkness) cycle; mean ± SEM values of triplicate plates, each with 30 parsnip mericarps, are presented. **c** Hormonal analysis of untreated and primed parsnip Panorama #3 mericarps in the dry state. The contents of gibberellin (GA) metabolites, abscisic acid (ABA) and its degradation products phaseic acid (PA) and dihydrophaseic acid (DPA), and of indole-3-acetic acid (IAA), *cis*-(+)−12-oxo-phytodienoic acid (*cis*-OPDA), and salicylic acid (SA) were quantified. Mean ± SEM values are presented, obtained from five biological replicates with ~ 100 fruits used per replicate sample

Germination assays with triplicate plates each with 50 carrot or 30 parsnip mericarps were performed in a Panasonic MLR-352 Environmental Test Chamber (Panasonic, Bracknell, UK) set to 20 °C (carrot cultivar Nerac, parsnip cultivars Panorama and Pacific #1) or 24 °C (carrot cultivar Newcastle) and continuous white light (~ 100 μmol s^−1^ m^−2^) as described earlier by Walker et al. (2025). Petri dishes (60 mm diameter, 15 mm height) with two filter papers (Machery-Nagel 713) were wetted with 2 ml deionised water (H_2_O). For experiments with parsnip cultivar Panorama #3 (Figs. 1b, c and 3d, e), a 30 °C/20 °C day/night (8 h light/16 h darkness) cycle was used. Germination was defined as the visible emergence of the radicle through all the encasing tissues. Dose–response germination assays were performed using gibberellin A_4+7_ (GA_4+7_; Duchefa Biochemie, Haarlem, The Netherlands) and *cis*,t*rans*-S(+)-abscisic acid (ABA, Duchefa) at the indicated concentrations. These hormones were added to the germination assays from concentrated stock solutions with either water or, for GA_4+7_, dimethyl sulfoxide (DMSO) as solvent; the DMSO concentrations used did not affect the germination curves (Walker et al. 2021). All treatments contained 0.1% plant preservative mixture PPM (Plant Cell Technology, Washington, USA). Maximal germination percentage (*G*_max_) and germination speed (Germination Rate GR_50%_ as the inverse of the time to reach 50% germination) were calculated from the germination curves as described (Loades et al. 2023; Steinbrecher et al. 2025b; Walker et al. 2025).

### Priming methods

The initial seed moisture content (SMC) of the mericarps (~ 5% per dry weight) was first determined by comparison of their fresh and dry weight using the Moisture analyzer (HB43-S Mettler Toledo). Five hundred milligrammes of mericarps were placed in opaque plastic priming drums (4 cm diameter; 60 ml volume) with the required water volume, achieving 50%, 60%, and 70% target SMC. The drums were constantly shaken at a speed of 8 rpm on the roller machine (Stuart roller mixer SRT9D) for 7 days or the duration indicated at 21 °C. At the end of the treatment, the mericarps were weighed and dried for 24 h at 26 °C. After drying back to the original SMC, primed mericarps were subjected to germination, embryo growth, and ageing assays. The embryo size of primed mericarps was measured initially with fully moistened seeds imbibed for 2 h and for the full time course of embryo growth over seven days. Additive priming with hormones was performed with 100 μM GA_4+7_ or 100 μM ABA for 7 days, and for ABA in addition for 14 days. Gas plasma-activated water (GPAW) using air as carrier gas was produced as described by Grainge et al. (2022b). Additive priming with GPAW was done for 2 days and 7 days, as indicated, as described in detail by Fatelnig et al. (2024).

### Artificial ageing assays

Chambers (Sainsbury’s Klip Lock storage boxes) with relative humidity (RH) of 70%, 80%, and 90% in the headspace above defined concentrations of lithium chloride (LiCl) solution were used (Hay et al. 2008). Wet ageing corresponds to seed storage conditions above 80% RH, whereas dry ageing corresponds to RH below 75% (Zinsmeister et al. 2020) or 60% (Hay et al. 2022). To conduct the wet ageing assays using Controlled Deterioration Treatment (CDT) (Fatelnig et al. 2024; Hourston et al. 2020; Chandler et al. 2020), triplicates of 50 mericarps were placed in Eppendorf tubes, which were resting on a rack in the headspace of the boxes above the LiCl solution (the availability of oxygen was therefore not limited), and incubated at the RH indicated for 3 or 7 days at 42 °C. After completion of wet ageing assays, mericarps were subjected to germination assays at 20 °C for ~ 2 (carrot) or ~ 3 (parsnip) weeks to quantify the reduction in germination vigour and ageing sensitivity compared to appropriate controls.

### Embryo growth assays and imaging

Internal embryo growth within imbibed mericarps was assessed at 20 °C and times indicated using the same incubation conditions as the germination assays. Approximately 50 mericarps were used per time point to measure sizes; they were longitudinally cut and photographed using a Leica DCF480 digital camera attached to a Leica Mz 12,5 stereomicroscope (Leica, Wetzlar, Germany) as described earlier (Walker et al. 2025). The embryo, seed and mericarp (seed plus pericarp) lengths were measured via the image analysis software ImageJ v1.6.0 (National Institute of Health, MA, USA). Embryo (E) sizes were represented as a ratio of the seed (S) length (E:S ratio) to account for the embryo-seed size association. Germinated mericarps were removed, and their values were replaced by a mean critical E:S ratio for radicle protrusion. The critical E:S ratio was calculated by measuring the internal embryo, seed and fruit lengths of 50 mericarps where the radicle had just protruded, as described in detail by Walker et al. (2025)

### Biomechanical measurements

Puncture force measurements were conducted as described previously using a modified custom-made biomechanics device (Steinbrecher and Leubner-Metzger 2017; Steinbrecher et al. 2025a, 2025b). In brief, a rounded metal pin with a 0.5 mm diameter was driven into the parsnip sample at 0.7 mm/min while force and displacement were recorded simultaneously. Parsnip mericarps were cut in half (split along the minor axis), placed in a 3D-printed sample holder with a cavity in the shape of a semi-ellipse, and the half not containing the embryo (distant-half endosperm) was measured.

### Plant hormone extraction and quantification

For whole mericarp quantification of gibberellins, abscisates, jasmonates, and auxins, Petri-dishes of mericarps were prepared as described for the germination assays. Five independent biological replicates were used for each time point, with approximately 100 mericarps used per replicate sample as described in detail by Walker et al. (2021). The levels of gibberellin (GA) metabolites, ABA, phaseic acid (PA), dihydrophaseic acid (DPA), indole-3-acetic acid (IAA), jasmonic acid (JA), jasmonoyl-L-isoleucine (JA-Ile), and *cis*-(+)−12-oxo-phytodienoic acid (*cis*-OPDA) were quantified by UHPLC-MS/MS as described (Flokova et al. 2014; Chandler et al. 2024; Simura et al. 2018; Walker et al. 2021; Urbanova et al. 2013).

## Results

### Hormones and embryo growth during hydropriming of morphologically dormant diaspores

**Fig. 2:**
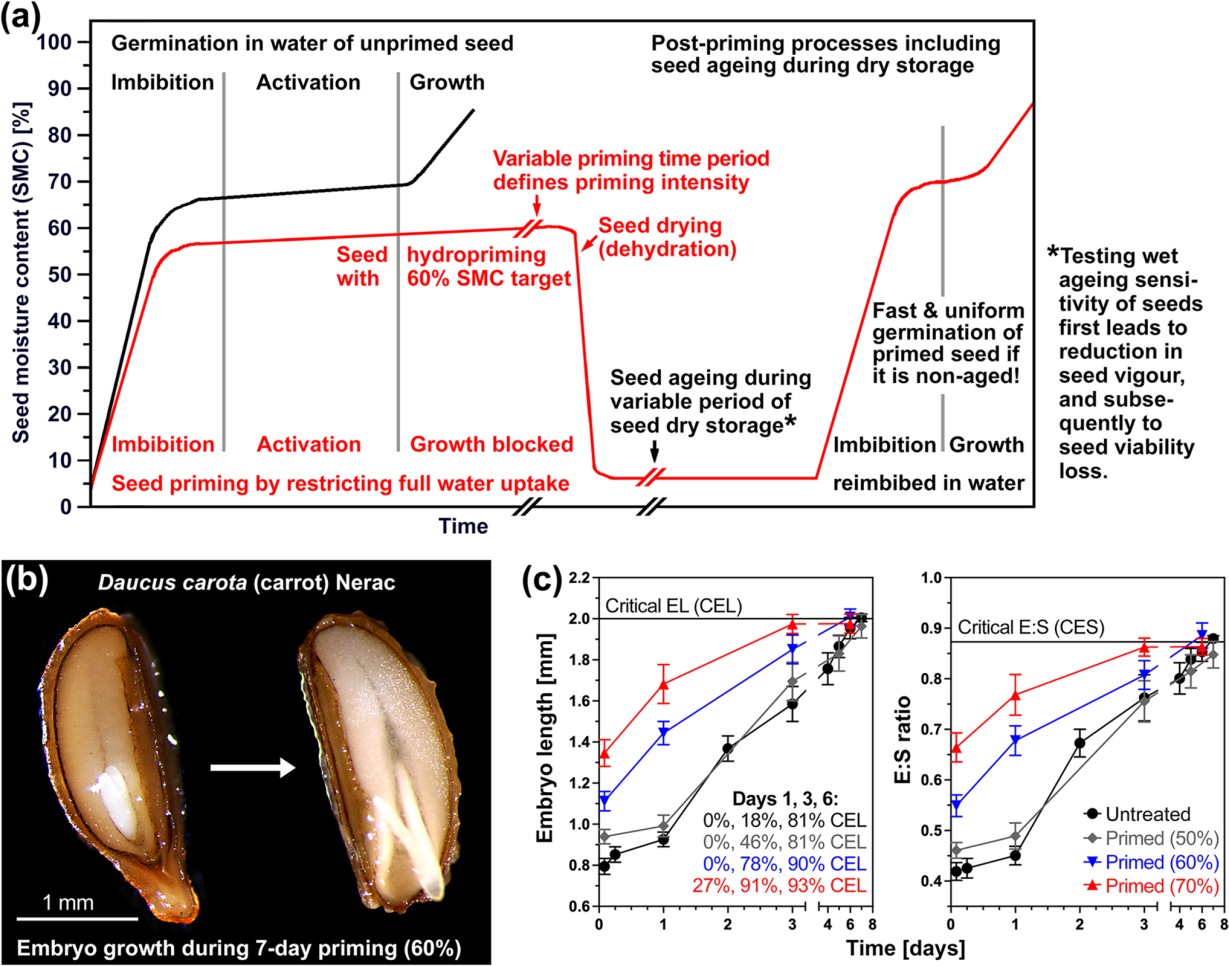
The Apiaceae hydropriming process and its effects on the pre-germination embryo growth inside carrot mericarps. **a** The hydropriming process as related to seed moisture content (SMC) and seed ageing during dry storage. During hydropriming using rotating drums, the defined limited addition of water allows imbibition and activation of the seed’s metabolism, but restricts full water uptake required for the completion of germination by radicle emergence and subsequent seedling growth. The seed drying step is an integral part of the seed priming process. Seed germination of primed seeds is fast and uniform. Seed ageing occurs during seed storage and causes vigour loss followed by viability loss, which can be experimentally quantified using ageing assays. **b** Pre-germination embryo growth inside carrot mericarps during hydropriming. **c** Carrot cultivar Nerac pre-germination embryo growth of imbibed mericarps, which were either not primed (untreated) or primed to three different target seed moisture contents: 50%, 60%, and 70%. The actual priming SMC ± SEM values obtained were 49.0 ± 0.3%, 59.0 ± 0.1%, 68.4 ± 0.2% for the Nerac, 50.5 ± 0.6%, 58.2 ± 0.2%, 67.6 ± 0.6% for the Pacific, and 49.2 ± 0.1%, 59.3 ± 0.1%, 68.5 ± 0.2% for the Panorama cultivar. Reaching the critical embryo length (CEL) and critical embryo:seed length ratio (CES) is required for the completion of germination by radicle emergence. For calculating the presented mean ± SEM values of ~ 50 embryos, only the embryo lengths ≤ CEL, i.e. the embryo lengths inside the mericarps, were considered. The percentages (*left panel*) of embryos which have reached the CEL at days 1, 3, 6 are indicated

The diaspores (dispersal units) of *Pastinaca sativa* (parsnip, Fig. 1), *Daucus carota* (carrot, Fig. 2), and other Apiaceae species are dry single-seeded indehiscent fruit halves called mericarps (Walker et al. 2021; Wojewodzka et al. 2019; Kadluczka and Grzebelus 2022). In a mericarp, the dead outer pericarp (fruit coat) and seed coat layers encase the abundant living endosperm tissue and the small (underdeveloped) embryo (Fig. 1a). A small embryo embedded in abundant living endosperm tissue is the hallmark of morphological dormancy (MD). In Apiaceae vegetable mericarps, the small embryo must first grow inside the imbibed mericarp from an initial embryo-seed (E:S) length ratio to the critical E:S ratio required for the completion of germination by radicle emergence (Fig. 1a). Figure 1 shows that the enhancement of germination by hydropriming of the parsnip cultivar Panorama is associated with pre-germination embryo growth inside the mericarp during the priming process. Hormone metabolite analysis demonstrated that hydropriming caused massive changes in the endogenous hormone contents of mericarps (Fig. 1c). This includes altered gibberellin (GA) metabolism in primed mericarps, with ~ 2.1-fold reduced contents of the bioactive GAs. Abscisic acid (ABA) degradation during priming results in ~ 9.2-fold reduced ABA contents, leading to a ~ 4.4-fold reduced ABA/GA ratio. The contents of indole-3-acetic acid (IAA), jasmonoyl-L-isoleucine (JA-Ile), *cis*-(+)−12-oxo-phytodienoic acid (*cis*-OPDA), and salicylic acid (SA) were reduced ~ 7.7, ~ 194, ~ 6.0, and ~ 4.7-fold, respectively (Fig. 1c). Parsnip mericarp priming was therefore associated with reductions in hormone contents, of which ABA and *cis*-OPDA are known germination inhibitors.

**Fig. 3:**
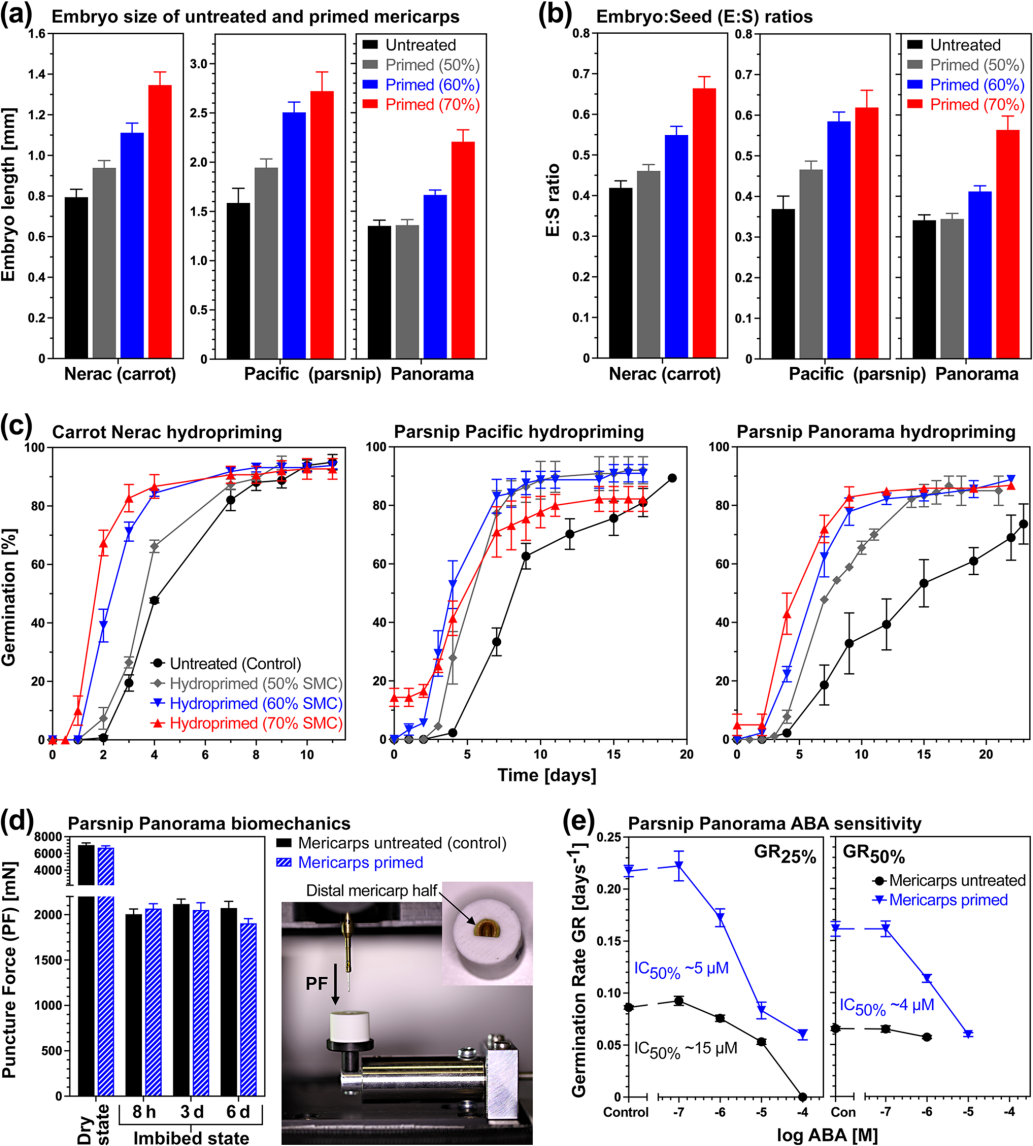
Comparative analysis of hydropriming effects on *Daucus carota* (carrot) and *Pastinaca sativa* (parsnip) embryo growth, biomechanics, and germination physiology. **a** The effect of different target seed moisture contents (SMC) on growth in embryo length during the 7 days of hydropriming of carrot cultivar Nerac and parsnip cultivars Pacific (lot #1) and Panorama (lot #1). **b** The effect of hydropriming with different target SMC on the embryo:seed (E:S) length ratios of carrot and parsnip cultivars; Mean ± SEM values of ~ 50 embryos. **c** The effect of hydropriming with different target SMC on the germination kinetics of carrot and parsnip cultivars at 20 °C in continuous white light. Mean ± SEM values of triplicate plates each with 50 carrot and 30 parsnip mericarps are presented. **d** The effect of hydropriming on the biomechanical weakening of the distal (embryoless) mericap half of parsnip cultivar Panorama (lot #3). Note the trend with reduced puncture force (PF) values for primed mericarps at day 6 during imbibition, which is however not significantly different to unprimed (untreated) mericarps. Mean ± SEM values of 30 mericarps are presented. **e** Comparative analysis of untreated and primed parsnip cultivar Panorama (lot #3) mericarp abscisic acid (ABA) sensitivity. Germination speed (GR_25%_ and GR_50%_) was calculated from the germination curves presented in Fig. 1b; mean ± SEM values are presented. Inhibition constants (IC_50%_) demonstrated that primed mericarps are ~ threefold more sensitive to ABA, but despite this, their germination speed remains higher compared to untreated mericarps across the entire ABA concentration range

Figure 2a summarises the hydropriming process, which was conducted as drum priming by adding a limiting amount of deionised water (H_2_O) to increase the seed moisture content (SMC) of the air-dry mericarps from the initial ~ 5% to the 50–70% target SMC. This imbibition allows activation of the metabolism and enhances pre-germination embryo growth, but the completion of germination by radicle emergence and post-germination embryo growth is blocked. After 7 days of drum priming at 20 °C, the parsnip and carrot mericarps were dried back to their original SMC, and either stored dry or analysed immediately for their germination and ageing tolerance (Fig. 2a). Pre-germination embryo growth inside mericarps during hydropriming of the carrot cultivar Nerac (Fig. 2b) was enhanced by increasing target SMCs, with 1/3 of the embryos having reached the critical E:S ratio already on day 1 with the 70% target SMC priming for which the actual priming SMC ± SEM was determined to be 68.4 ± 0.2% (Fig. 2c). Similar enhancements in pre-germination embryo growth with increasing target SMC were obtained for the parsnip cultivars Pacific and Panorama (Fig. 3a, b), but 70% target SMC (see legend of Fig. 3 for actual measured SMC) caused overpriming resulting in 14.4% and 4.9%, respectively, germinated mericarps already during the priming treatment. Figure 3c demonstrates that hydropriming enhances the germination rates (GR) up to ~ twofold for GR_50%_, that is the inverse of the population’s time to reach 50% germination. While this was accompanied by pre-germination embryo growth during the priming treatment (Fig. 3a), which enlarges the embryo cavity in the embryo-half endosperm (Figs. 1a, 2b), it was not associated with significant weakening of the distant-half endosperm and pericarp (Fig. 3d). Parsnip mericarp priming was not only associated with reduced ABA contents and increased germination speed, but also with altered ABA sensitivity of germination (Figs. 1b, 3e).

### Effects of additive priming with GA and ABA on embryo growth and germination

**Fig. 4:**
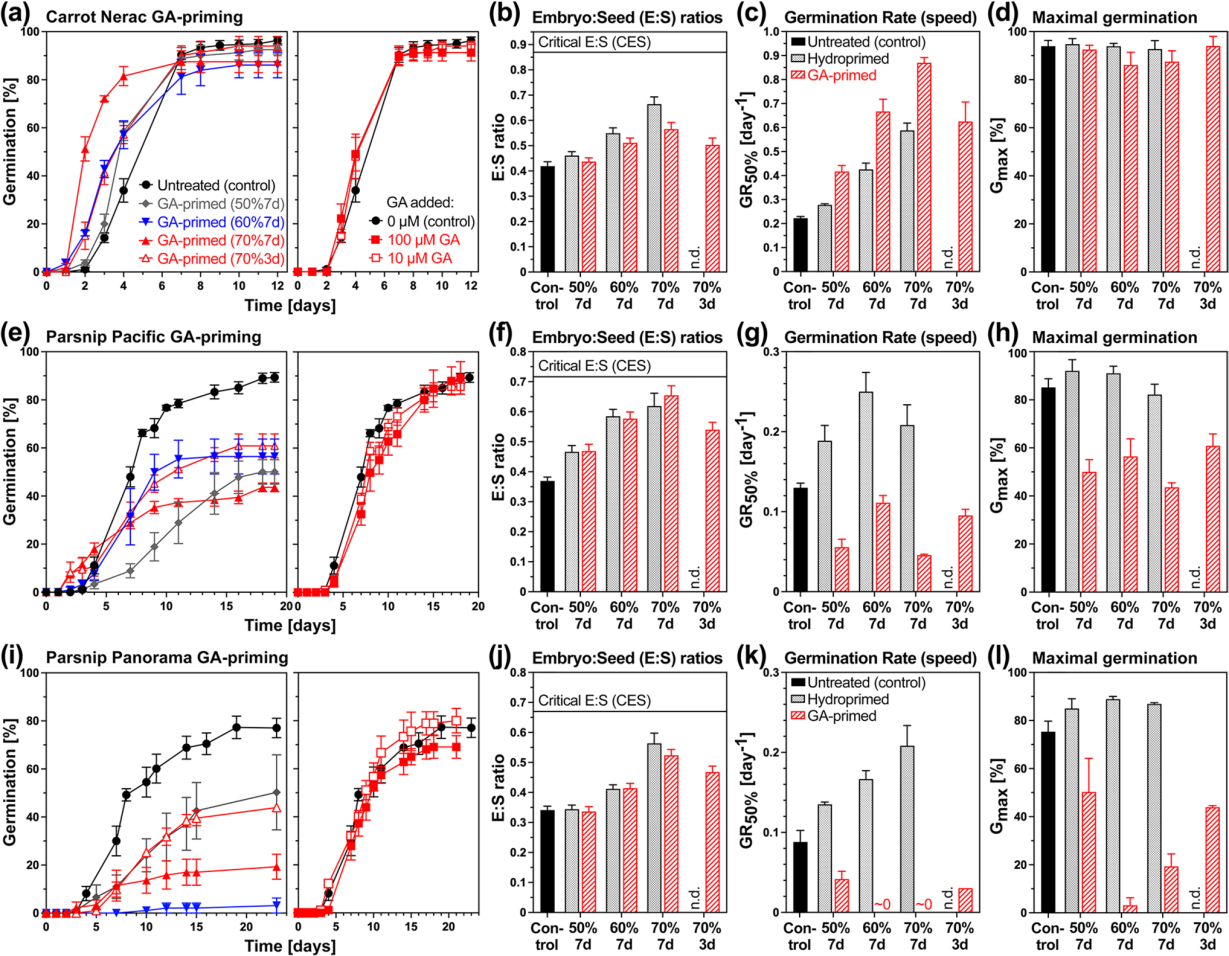
Comparative analysis of priming with gibberellins (GA) on *Daucus carota* (carrot) and *Pastinaca sativa* (parsnip) on embryo growth and germination. **a** The effect of different target seed moisture contents (SMC) during 3 or 7 days GA-priming with 100 µM GA_4+7_ (*left panel*) on the germination of carrot cultivar Nerac mericarps. Mean ± SEM values of triplicate plates each with 50 mericarps imbibed at 20 °C in continuous white light are presented. For comparison, germination of unprimed mericarps without and with 10 and 100 µM GA_4+7_ (*right panel*) is presented. **b** The effect of GA priming versus hydropriming with different target SMC on the embryo:seed (E:S) length ratios of the carrot Nerac cultivar; Mean ± SEM values of ~ 50 embryos; n.d., not determined. The critical E:S ratio (CES) required for the completion of germination by radicle protrusion is indicated as a grey line. **c** The effect of GA priming versus hydropriming on the germination rate (speed) GR_50%_, i.e. the inverse of the time required to reach 50% germination, of carrot cultivar Nerac; Mean ± SEM values calculated from the germination curves (Figs. 4a and 3c). **d** The effect of GA priming versus hydropriming on the maximal germination percentage *G*_max_ of carrot cultivar Nerac; Mean ± SEM values (from Figs. 4a and 3c). **e** Analysis of GA-priming with 100 µM GA_4+7_ (*left panel*) and GA addition (*right panel*) on the germination of parsnip cultivar Pacific mericarps (lot #1). Mean ± SEM values of triplicate plates each with 30 mericarps imbibed at 20 °C in continuous white light are presented. **f** The effect of GA priming on the E:S length ratios of the parsnip Pacific cultivar; Mean ± SEM values of ~ 50 embryos. **g** The effect of GA priming on the germination speed GR_50%_ of parsnip cultivar Pacific; Mean ± SEM values (from Fig. 4e). **h** The effect of GA priming on *G*_max_ of parsnip cultivar Pacific; Mean ± SEM values (from Fig. 4e). **i** Analysis of GA-priming with 100 µM GA_4+7_ (*left panel*) and GA addition (*right panel*) on the germination of parsnip cultivar Panorama mericarps (lot #1). **j** The effect of GA priming on the E:S length ratios of the parsnip Panorama cultivar. **k** The effect of GA priming on the germination speed GR_50%_ of parsnip cultivar Panorama; Mean ± SEM values (from Fig. 4i). **l** The effect of GA priming on *G*_max_ of parsnip cultivar Panorama. Note that the GR_50%_ and *G*_max_ values of corresponding hydroprimed parsnip mericarps were derived from Fig. 3c

**Fig. 5:**
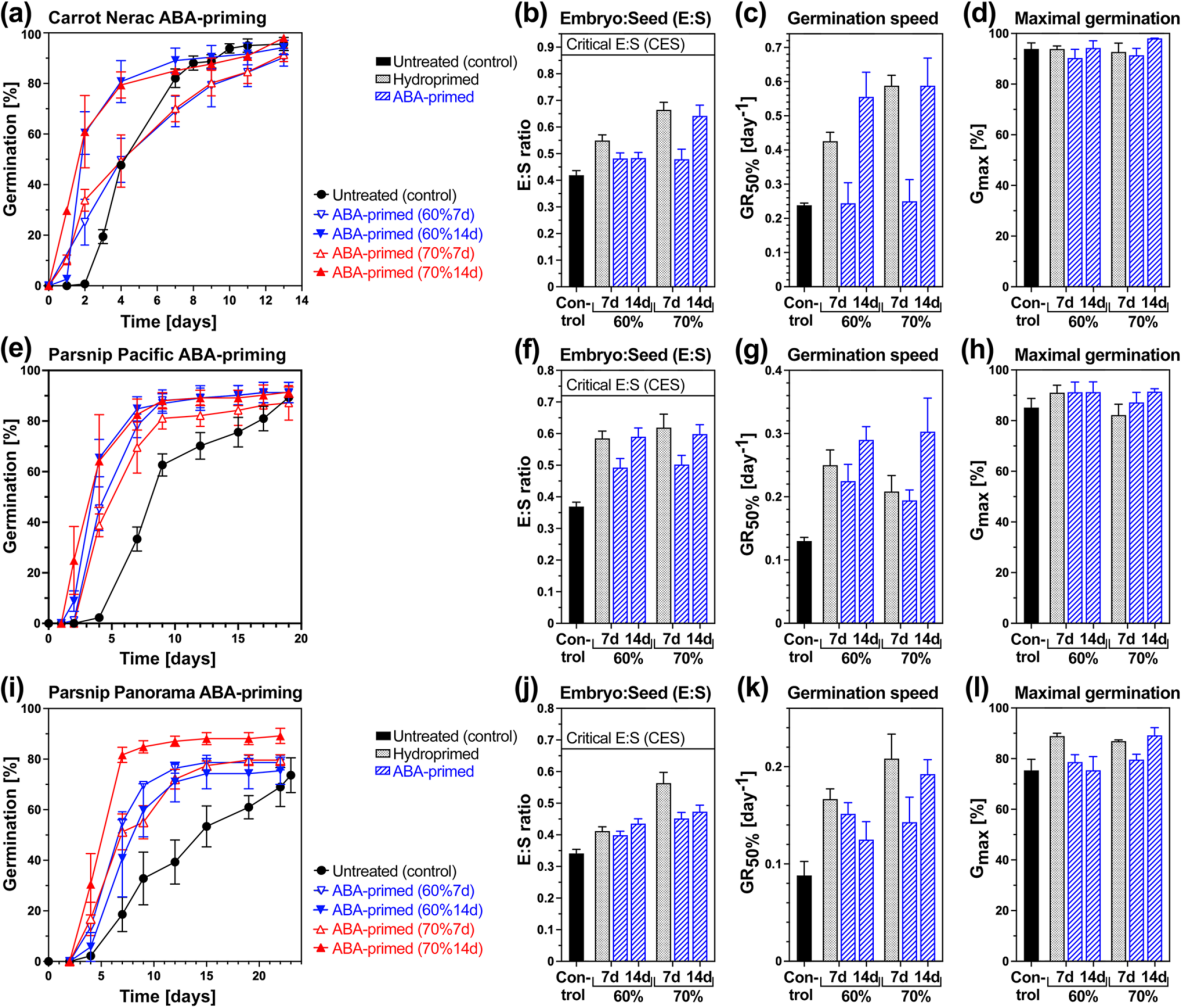
Comparative analysis of priming with abscisic acid (ABA) on *Daucus carota* (carrot) and *Pastinaca sativa* (parsnip) on embryo growth and germination. **a** The effect of different target seed moisture contents (SMC) during 7 or 14 days ABA-priming with 100 µM ABA on the germination of carrot cultivar Nerac mericarps. Mean ± SEM values of triplicate plates each with 50 mericarps imbibed at 20 °C in continuous white light are presented. **b** The effect of ABA priming versus hydropriming with different target SMC on the embryo:seed (E:S) length ratios of the carrot Nerac cultivar; Mean ± SEM values of ~ 50 embryos. The critical E:S ratio (CES) required for the completion of germination by radicle protrusion is indicated as a grey line. **c** The effect of ABA priming versus hydropriming on the germination rate (speed) GR_50%_, i.e. the inverse of the time required to reach 50% germination, of carrot cultivar Nerac; Mean ± SEM values calculated from the germination curves (Figs. 5a and 3c). **d** The effect of ABA priming versus hydropriming on the maximal germination percentage *G*_max_ of carrot cultivar Nerac; Mean ± SEM values (from Figs. 5a and 3c). **e** Analysis of ABA-priming with 100 µM ABA on the germination of parsnip cultivar Pacific mericarps (lot #1). Mean ± SEM values of triplicate plates each with 30 mericarps imbibed at 20 °C in continuous white light are presented. **f** The effect of ABA priming on the E:S length ratios of the parsnip Pacific cultivar; Mean ± SEM values of ~ 50 embryos. **g** The effect of ABA priming on the germination speed GR_50%_ of parsnip cultivar Pacific; Mean ± SEM values (from Fig. 4e). **h** The effect of ABA priming on *G*_max_ of parsnip cultivar Pacific; Mean ± SEM values (from Fig. 4e). **i** Analysis of ABA-priming with 100 µM ABA on the germination of parsnip cultivar Panorama mericarps (lot #1). **j** The effect of ABA priming on the E:S length ratios of the parsnip Panorama cultivar. **k** The effect of ABA priming on the germination speed GR_50%_ of parsnip cultivar Panorama; Mean ± SEM values (from Fig. 4i). **l** The effect of ABA priming on *G*_max_ of parsnip cultivar Panorama. Note that the GR_50%_ and *G*_max_ values of corresponding hydroprimed parsnip mericarps were derived from Fig. 3c.

Additive priming with hormones was achieved by adding 100 µM GA_4+7_ (Fig. 4) or 100 µM ABA (Fig. 5) during the priming treatment as compared to the corresponding hydropriming (Figs. 3c, 4, 5). In contrast to GA addition to the imbibition medium for germination, GA-priming enhanced the germination of the Nerac carrot cultivar (Fig. 4a). GA-priming did not act by enhancing embryo growth beyond the hydropriming values (Fig. 4b), but it enhanced the speed of germination (GR_50%_) well above the GR_50%_ values obtained by hydropriming (Fig. 4c). While this enhancement of the germination speed was associated with pre-germination embryo growth, the observed embryo growth was somewhat lower in GA-primed compared to hydroprimed mericarps (Fig. 4b). GA-priming also slightly reduced the maximal germination percentages (*G*_max_) of carrot mericarps (Fig. 4d). In contrast to carrot, GA-priming of the parsnip cultivars Pacific (Fig. 4e–h) and Panorama (Fig. 4i–l) drastically reduced GR_50%_ and *G*_max_ to ≤ 50% of the hydroprimed and untreated values. This was not associated with reduced pre-germination embryo growth, as this was very similar between GA-primed and hydroprimed parsnip mericarps (Fig. 4f, j). In contrast to GA-priming, which had positive (carrot) or negative (parsnip) effects, the addition of 10–100 µM GA_4+7_ to the imbibition medium of unprimed mericarps did not appreciably affect the speed (GR_50%_) and *G*_max_ of carrot and parsnip germination (Fig. 4).

Addition of 1–100 µM ABA to the imbibition medium of parsnip mericarps inhibited germination in a dose-dependent manner (Fig. 1b), and 100 µM ABA inhibits carrot germination (Homrichhausen et al. 2003). In contrast to this, additive priming with 100 µM ABA promoted carrot and parsnip germination compared to unprimed control (Fig. 5a, e, i). Drum hydropriming for 7 days compared to ABA-priming for 7 (ABA7d) or 14 (ABA14d) days revealed that ABA14d resulted in roughly equal or higher germination speed (GR_50%_) compared to hydropriming, while ABA7d delivered in general GR_50%_ values between the values for unprimed and hydroprimed mericarps (Fig. 5c, g, k). Compared to hydroprimed mericarps, ABA-priming did not appreciably affect the *G*_max_ values which were equal or above the *G*_max_ values of unprimed mericarps. Pre-germination embryo growth during ABA-priming occurred well above the control values and was for ABA14d, roughly similar to the hydropriming values (Fig. 5b, f, j). Hydropriming for 7 days with 70% target SMC can cause over-priming (Fig. 3). In contrast to this, ABA-priming with 70% target SMC for 7 or 14 days did not cause over-priming (Fig. 5). ABA-priming is therefore a suitable technology for achieving maximum priming intensities of carrot and parsnip without the risk of over-priming.

### Increased ageing tolerance of ABA-primed compared to hydroprimed Apiaceae mericarps

**Fig. 6:**
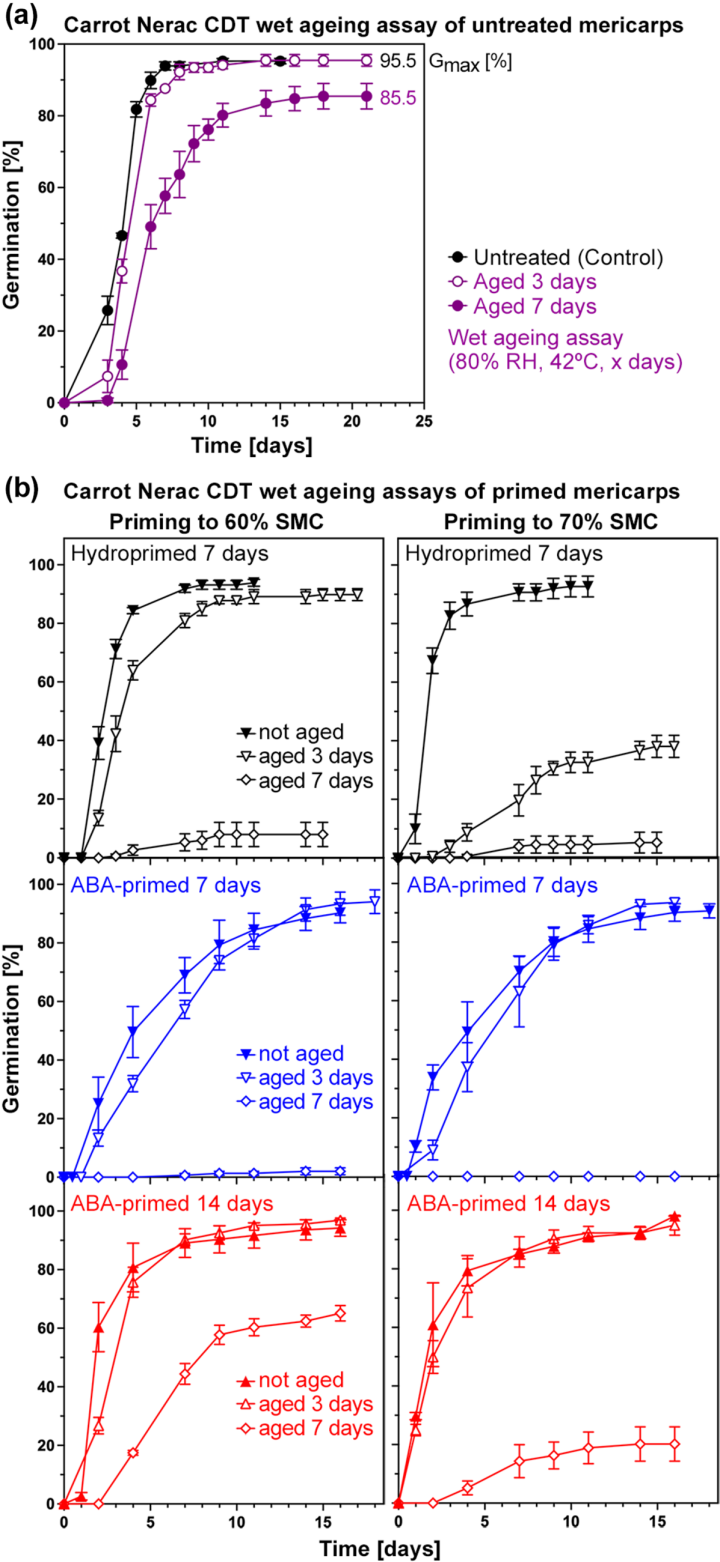
Comparative analysis of vigour and ageing sensitivity of untreated, hydroprimed, and ABA-primed *Daucus carota* (carrot) cultivar Nerac using the wet ageing assay. **a** Germination analysis (imbibed at 20 °C in continuous white light) of untreated Nerac mericarps after conducting the wet ageing assay by incubation at 42 °C at 80% relative humidity (RH) for 3 or 7 days. Mean ± SEM values of triplicate plates each with 50 mericarps; *G*_max_, maximal germination percentage. **b** Germination analysis of hydroprimed (7 days to 60% or 70% seed moisture content (SMC)) and ABA-primed (7 days or 14 days to 60% or 70% SMC) Nerac mericarps after conducting the CDT ageing assay (42 °C, 80% RH, 3 or 7 days). Mean ± SEM values of triplicate plates each with 50 mericarps

**Fig. 7:**
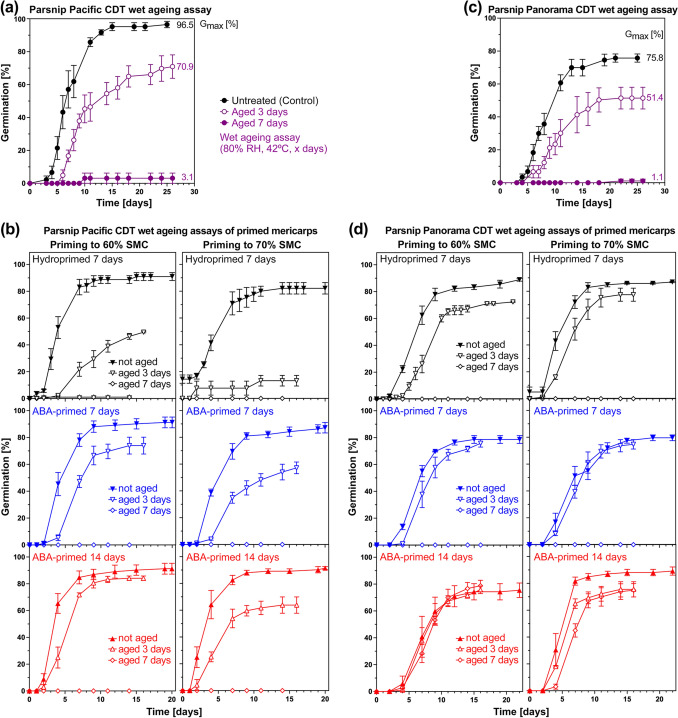
Comparative analysis of vigour and ageing sensitivity of untreated, hydroprimed, and ABA-primed *Pastinaca sativa* (parsnip) cultivars Pacific and Panorama using the wet ageing assay. **a** Germination analysis (imbibed at 20 °C in continuous white light) of untreated Pacific mericarps (lot #1) after conducting the wet ageing assay by incubation at 42 °C at 80% relative humidity (RH) for 3 or 7 days. Mean ± SEM values of triplicate plates each with 30 mericarps; *G*_max_, maximal germination percentage. **b** Germination analysis of hydroprimed (7 days to 60% or 70% seed moisture content (SMC)) and ABA-primed (7 days or 14 days to 60% or 70% SMC) Pacific mericarps after conducting the wet ageing assay (42 °C, 80% RH, 3 or 7 days). **c** Germination analysis (imbibed at 20 °C in continuous white light) of untreated Panorama mericarps (lot #1) after conducting the wet ageing assay. **d** Germination analysis of hydroprimed and ABA-primed Panorama mericarps after conducting the wet ageing assay. Mean ± SEM values of triplicate plates each with 30 mericarps

**Fig. 8:**
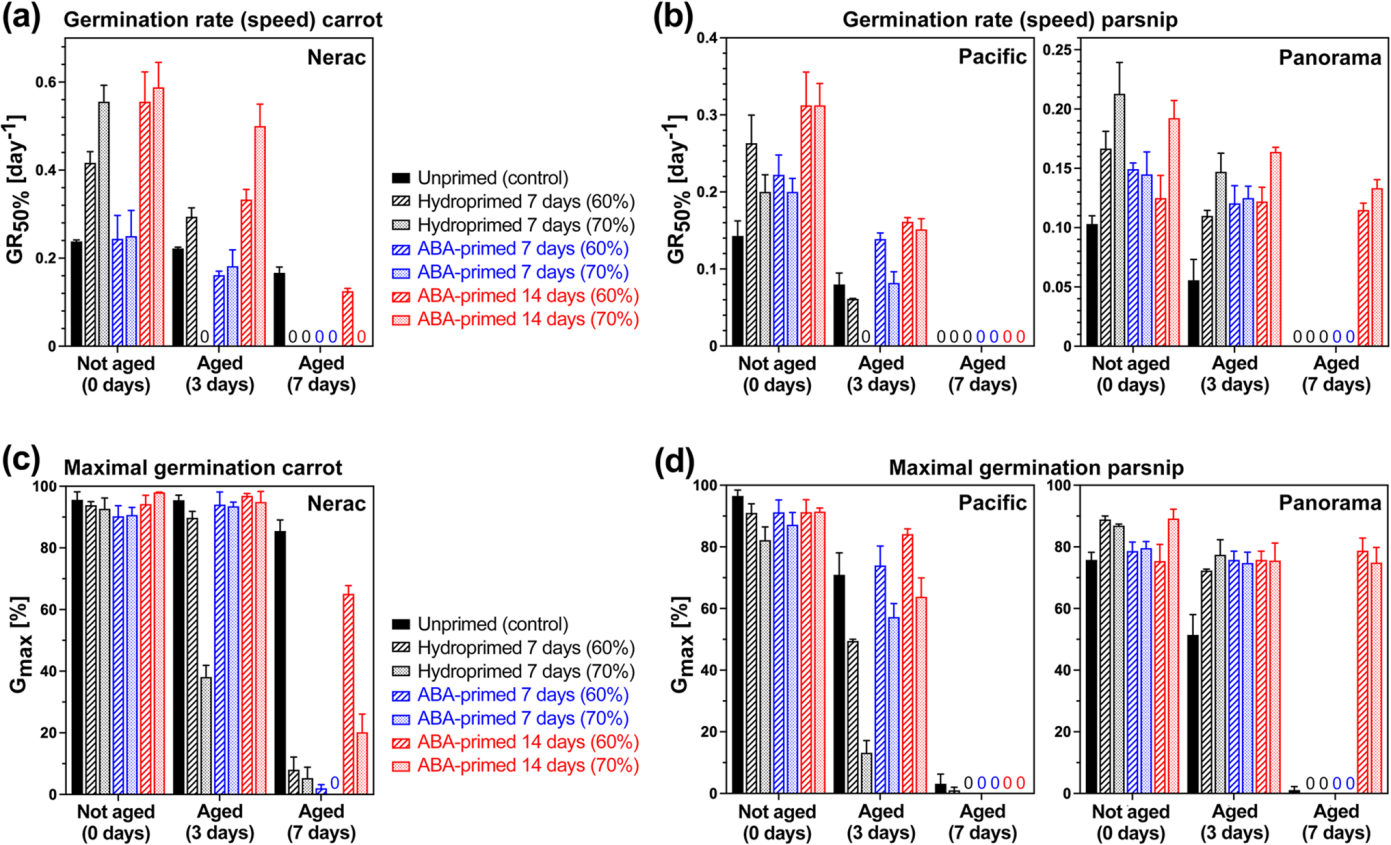
Comparative analysis using the wet ageing assay of *Daucus carota* (carrot) and *Pastinaca sativa* (parsnip) mericarp priming on the germination speed (GR_50%_) and maximal germination percentages (*G*_max_). **a** Quantified wet ageing sensitivity effects on carrot cultivar Nerac mericarp germination rates GR_50%_, i.e. the inverse of the time required to reach 50% germination. Hydropriming for 7 days (60% and 70% target seed moisture content (SMC)) and ABA-priming with 100 µM ABA for 7 or 14 days (60% and 70% target SMC); Mean ± SEM values calculated from the germination curves (Fig. 6). **b** Quantified wet ageing sensitivity effects on parsnip cultivars Pacific and Panorama mericarp germination rates GR_50%_. Hydropriming for 7 days (60% and 70% target SMC) and ABA-priming with 100 µM ABA for 7 or 14 days (60% and 70% target SMC); Mean ± SEM values calculated from the germination curves (Fig. 7). **c** Quantified wet ageing sensitivity effects on carrot cultivar Nerac mericarp maximal germination percentages (*G*_max_). **d** Quantified wet ageing sensitivity on parsnip cultivars Pacific and Panorama mericarp *G*_max_); Mean ± SEM values

Artificial assays can be used to quantify seed vigour and ageing sensitivity during dry or wet storage (Bruggink et al. 1999; Hay 2022; Zinsmeister et al. 2020; Hay et al. 2019; Corbineau 2024). To establish suited ageing assays to quantify vigour changes for carrot and parsnip, we incubated their mericarps for 3 and 7 days at 42 °C and 70–90% relative humidity (RH) followed by analysing germination (Fig. S1). The wet ageing assay with 80% RH provided the best conditions for comparatively analysing the ageing sensitivities of untreated (control), hydroprimed, and ABA-primed carrot (Fig. 6) and parsnip (Fig. 7) mericarps. The priming was with 60% and 70% target SMC (Figs. 6, 7 and 8), but for carrot and for hydropriming of both species also 50% target SMC was investigated (Fig. S2). For the carrot cultivar Nerac, hydropriming (Fig. 6b) increased the ageing sensitivity compared to the control (Fig. 6a), which resulted in reduced germination speed (GR_50%_, Fig. 8a) and *G*_max_ (Fig. 8b) of hydroprimed mericarps compared to the control. ABA-priming, in particular if conducted for 14 days (ABA14d), partially or fully reverted the increased ageing sensitivity of primed mericarps (Figs. 6b, 8a, c). ABA-priming retained their vigour and thereby increased their ageing tolerance, leading to GR_50%_ values of 3-day aged ABA14d-primed mericarps similar to the unaged hydroprimed mericarps, and to high *G*_max_ values similar to unaged mericarps. Similar results were obtained for the parsnip cultivars Pacific and Panorama (Figs. 7, 8), the wet ageing assay results demonstrated also here that ABA14d-primed mericarps had increased ageing tolerance and retained vigour. Panorama mericarps’ ageing tolerance was generally higher than Pacific mericarps, which was in particular pronounced for ABA14d-primed mericarps (Figs. 7b, d, 8b, d). ABA-priming for 14 days therefore seems to be an excellent method to mitigate the negative effects of hydropriming on carrot and parsnip mericarp vigour and wet ageing sensitivity.

### Priming with gas plasma-activated water to enhance germination performance with retaining seed ageing resilience of Apiaceae and other vegetables

**Fig. 9:**
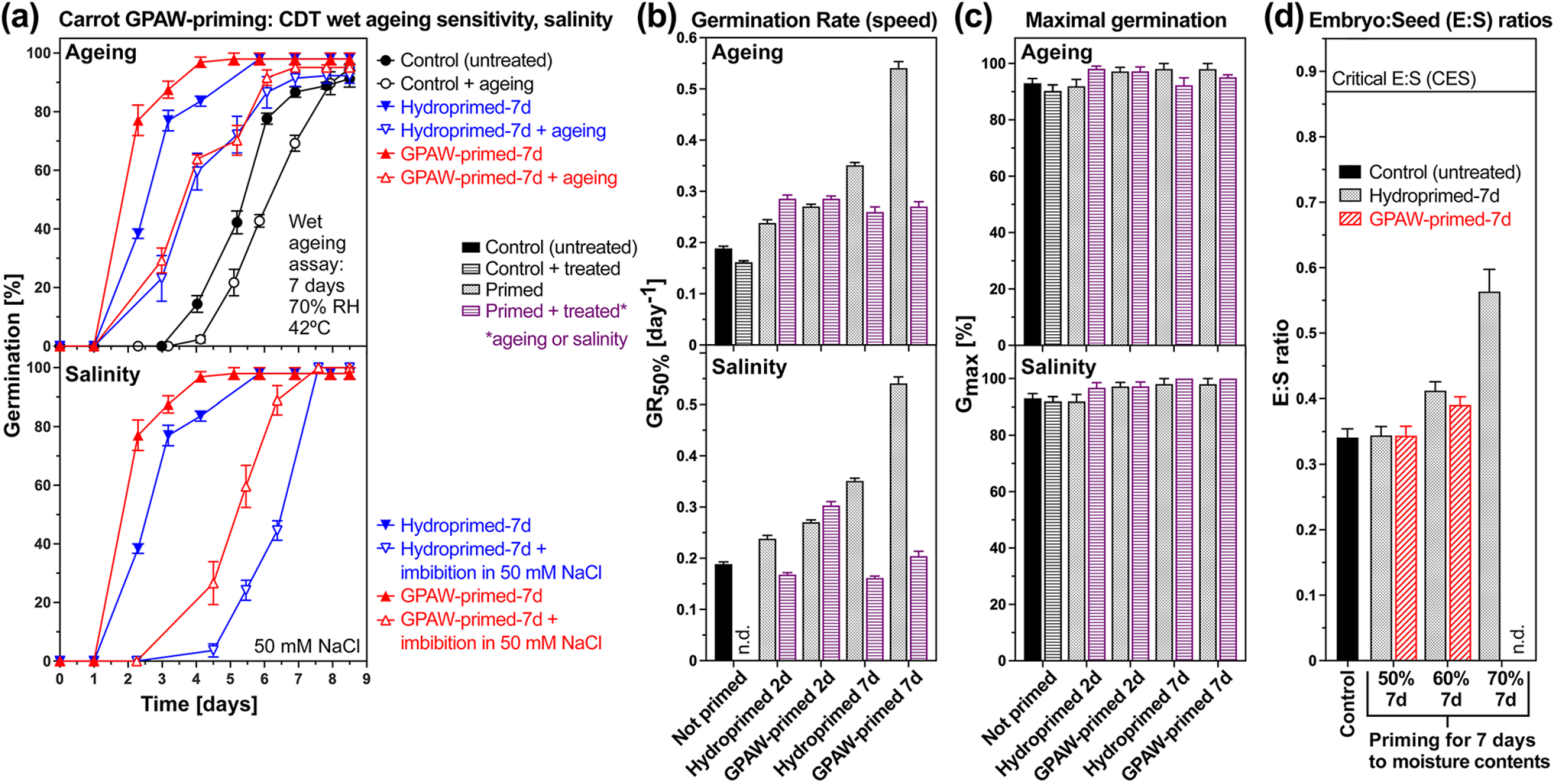
Analysis of priming with gas plasma-activated water (GPAW) on germination, embryo growth, ageing, and salinity resilience of *Daucus carota* (carrot) mericarps. **a** The effect of GPAW-priming (7 days) and hydropriming (7 days) on germination, ageing sensitivity (*upper panel*), and salinity resilience (*lower panel*) of carrot cultivar Newcastle. The wet ageing assay, that is, incubation of mericarps at 42 °C, 70% relative humidity (RH) for 7 days, was used to estimate ageing sensitivity (*upper panel*). Germination in the presence of 50 mM NaCl was used to estimate salinity resilience (*lower panel*). Mean ± SEM values of triplicate plates, each with 30 mericarps, are presented. **b** The effect of GPAW-priming and hydropriming for 2 days or 7 days on the germination rate (speed) GR_50%_, i.e. the inverse of the time required to reach 50% germination; Mean ± SEM values calculated from the germination curves (Fig. 9a). **c** The effect of GPAW-priming and hydropriming on the maximal germination percentage *G*_max_; Mean ± SEM values (from Fig. 9a). **d** The effect of GPAW-priming and hydropriming for 7 days with different target seed moisture contents (SMC) on the embryo:seed (E:S) length ratios of the carrot Nerac cultivar; Mean ± SEM values of ~ 50 embryos. The critical E:S ratio (CES) required for the completion of germination by radicle protrusion is indicated as a grey line

Recent advances in seed priming techniques (Bruggink 2022) include the gas plasma-activated water (GPAW) additive priming technology, which can enhance germination performance while retaining seed ageing resilience and vigour (Fatelnig et al. 2024; Grainge et al. 2021). The GPAW produced for this study was standardised using a bubble reactor with air as the carrier gas, and the quantified active chemical species generated (Fig. S3) were very similar to our earlier work (Grainge et al. 2022b). GPAW-priming of mericarps of the carrot cultivar Newcastle for 7 days (Fig. 9a) or 2 days (Fig. S4) demonstrated that it is better compared to hydropriming by providing increased germination speed (GR_50%_, Fig. 9b) when imbibed for germination in water or in 50 mM NaCl (salinity). GPAW-primed carrot mericarps therefore have an increased salinity tolerance compared to hydroprimed carrot mericarps. Figure 9d shows that pre-germination embryo growth is similarly enhanced by GPAW-priming and hydropriming, which suggests that the observed increase in germination speed by GPAW-priming compared to hydropriming (Fig. 9a) is not achieved through GPAW promotion of the pre-germination embryo growth in MD diaspores. The ageing tolerance of GPAW-primed carrot mericarps was similar to hydroprimed mericarps (Fig. 9) with only a slightly increased (~ 1.1-fold) estimated storability (Suppl. Tables 2 and 3).

GPAW-priming also enhanced the germination of diaspores with fully developed embryos, including such as the cereal grains of the crop tef (Fatelnig et al. 2024), lettuce fruits (Fig. S5), beetroot fruits, and various Brassicaceae seeds (Suppl. Table 2). In contrast to carrot mericarps, their estimated increased storability by GPAW-priming compared to hydropriming was ~ threefold for lettuce (Fig. S5) and tef (Suppl. Table 2), ~ 1.5-fold for the Brassicaceae, and ~ twofold for the Amaranthaceae vegetables (Suppl. Table 2). It therefore seems that while GPAW-priming enhanced the germination speed beyond hydropriming for all species, including carrot, the additional beneficial retainment of diaspore ageing resilience by GPAW-priming observed for diaspores with a fully developed embryo was not observed for carrot, representing Apiaceae MD diaspores with an underdeveloped (small) embryo.

## Discussion

### Hydropriming of Apiaceae mericarps has pre-germination embryo growth and hormonal regulation as targets

We demonstrate here that hydropriming of carrot and parsnip mericarps causes pre-germination embryo growth inside the seed at the expense of the endosperm (Figs. 1, 2, 3, 4 and 5). The relative embryo size expressed as embryo:seed (E:S) length ratios increased with increased priming target SMC. At the highest priming intensity (70% SMC), hydropriming caused a ~ 1.6-fold increase in carrot and parsnip embryo size (E:S ratio), while a ~ 2.1-fold (carrot) and ~ 1.8-fold (parsnip) increase is required to reach the critical E:S ratio (CES). Reaching the CES is a requirement for the completion of germination of Apiaceae mericarps and is normally achieved by imbibition for germination with unlimited water availability (Homrichhausen et al. 2003; Walker et al. 2021). In contrast to seed priming of species with fully developed embryos (Corbineau et al. 2023; Gran et al. 2025; Pagano et al. 2023), enhancing pre-germination embryo growth is therefore a developmental hallmark of the seed priming technologies, as demonstrated by embryo length measurements in the Apiaceae vegetable crops carrot (this work, Dawidowicz-Grzegorzewska 1997), parsnip (this work, Gray et al. 1984), celery (Van der Toorn and Karssen 1992; Van der Toorn 1990; Thomas 1984), and parsley (Olszewski et al. 2004, 2005). The works on parsley also demonstrated that not only embryo length but also embryo volume increased for the cotyledons and the hypocotyl/radicle, as well as that the endosperm volume decreased and, after longer priming duration, pericarp degradation was noted. In agreement with this, we found that the endosperm cavity required for the priming-enlarged carrot and parsnip embryo was increased (Figs. 1, 2) and that there is a possible trend towards decreased biomechanical strength upon priming (Fig. 3d). The observed increasing enhancement of the germination speed with increasing priming intensity of carrot and parsnip (Fig. 3) was, without quantifying embryo growth, also observed in priming work with diverse Apiaceae crops (Brocklehurst and Dearman 1983b, a; Corbineau et al. 2023; Zhao et al. 2021; Olszewski et al. 2005; Rahimi 2013). Subsequent seedling growth and, in some cases, harvest yield was also enhanced by the priming treatment of Apiaceae crops (Brocklehurst and Dearman 1983b; Mirshekari 2012; Mahmood-ur-Rehman et al. 2024).

While seed priming of Apiaceae vegetable crops has enhanced pre-germination growth of the underdeveloped (small) embryo as a major target (*see previous paragraph*), priming of crop seeds such as tomato, lettuce, brassicas, sugar beet, sunflower, cereals, and *Arabidopsis thaliana* don’t have this requirement as they have fully developed embryos (Baskin and Baskin 2014; Carta et al. 2024; Finch-Savage and Leubner-Metzger 2006; Steinbrecher and Leubner-Metzger 2017; Gran et al. 2025; Argerich et al. 1989; Chojnowski et al. 1997). Although it is assumed that changes in the seed’s hormonal network during priming resemble those during early imbibition, this hypothesis has been rarely verified by direct hormone quantification in seeds with fully developed embryos. The few examples available include that during tomato hydropriming Nakaune et al. (2012) reported a ~ 2.2-fold increase in the seed ABA content, while Garcia et al. (2021) reported a ~ 1.3-fold decrease in ABA and a ~ 1.2-fold increase in GA contents in seeds. For tomato osmopriming, El-Araby et al. (2006) reported a ~ 1.9-fold ABA decrease, a ~ 1.6-fold GA_3_ increase, and a ~ 1.5-fold IAA decrease in the seed contents. For soybean seed hydropriming, a ~ 1.2-fold decrease in ABA and a ~ 1.5-fold increase in GA_4_ contents (Manoharlal and Saiprasad 2019), and for tobacco seed hydropriming a ~ 1.2-fold decrease in ABA and IAA, and no increase in GA_1_ or GA_3_ contents was measured (Zhang et al. 2018). For barley, hydropriming decreased the ABA contents ~ 1.6-fold (Gendreau et al. 2008), and for rice, hydropriming decreased the ABA contents ~ 1.5-fold and increased the GA contents ~ 1.3-fold (Sheteiwy et al. 2017). Taken together, these examples demonstrate that the observed changes in ABA, GA, and IAA contents in seeds with fully developed embryos seem to be relatively small.

In contrast to this, we observed ~ 9.2-fold reduced ABA, ~ 7.7-fold reduced IAA, and ~ 2.1-fold reduced contents in bioactive GAs (Fig. 1). This leads to a ~ 4.4-fold reduced ABA/GA ratio and a ~ 3.7-fold reduced ABA/IAA ratio in primed parsnip mericarps (Fig. 1). While the bioactive GAs are mainly localised in the embryo, the ABA is mainly in the pericarp and endosperm and is rapidly degraded during early germination (Walker et al. 2021, 2025; Nakabayashi et al. 2025). While 100 µM ABA itself does not inhibit carrot, parsnip, and celery pre-germination embryo growth (this work, Homrichhausen et al. 2003; Walker et al. 2021), the dynamic changes in the ABA/GA and ABA/IAA ratios in Apiaceae mericarps are important for the pre-germination embryo growth, endosperm degradation, and subsequent pericarp/endosperm rupture to complete germination. Further to this, jasmonoyl-L-isoleucine (JA-Ile), *cis*-(+)−12-oxo-phytodienoic acid (*cis*-OPDA), and salicylic acid (SA) were reduced ~ 194, ~ 6.0, and ~ 4.7-fold, respectively (Fig. 1c). The *cis*-OPDA is mainly localised in the parsnip pericarp (Nakabayashi et al. 2025) and is known for its control of *Arabidopsis thaliana* seed dormancy (Dave et al. 2016) and for maintaining sunflower dormancy by counteracting the dormancy-breaking effects of cytokinins (Del Bel et al. 2023). Primed parsnip mericarps not only altered in their hormone contents but also were affected in their ABA sensitivity (Figs. 1, 3e), without being affected in their GA sensitivity (Fig. 4). Together with the changes in hormone contents, this supports the conclusion that priming interferes with the network of hormonal interactions to provide the enhanced germination performance.

### Additive priming with phytohormones enhances germination without beyond-hydropriming enhanced pre-germination embryo growth

Additive priming with phytohormones (hormopriming) has emerged as an important tool not only for enhancing germination, but beyond this for increasing abiotic stress tolerance of seedlings and established crop plants (Rhaman et al. 2021; Rawal et al. 2024; Pagano et al. 2023). Additive seed priming with GA, often as 50 µM to > 1 mM GA_3_, for example, increased the salinity and drought tolerance of legumes, cereals, brassicas, and lettuce, and in some cases also increased yield production (Rhaman et al. 2021; Ibrahim 2016; Zhu et al. 2021). Tobacco seed GA-priming with 250 µM GA_3_ resulted in ~ 1.3-fold decreased ABA and IAA contents, while the seed GA_3_ contents were increased from zero to ~ 2 µmol/g dry weight (Zhang et al. 2018). Transcriptome analysis revealed that, compared to hydropriming, the GA-priming was, amongst others, associated with down-regulation of ABA biosynthesis genes and auxin response factors, suggesting that GA affected the ABA and IAA/auxin interactions in these seeds with fully developed embryos.

GA-priming enhanced the germination of fennel and parsley mericarps under salinity and heat stress (Pill and Kilian 2000; Attia et al. 2022), but the effects on the growth of the underdeveloped embryo were not investigated in these Apiaceae species. We found that GA-priming with 100 µM GA_4+7_ enhanced carrot germination speed (GR_50%_) without appreciably affecting maximal germination (*G*_max_), but it severely reduced parsnip GR_50%_ and *G*_max_ (Fig. 4). Interestingly, neither the enhancement of carrot germination speed nor the reduction in parsnip germination speed and *G*_max_ were associated with appreciable changes in pre-germination embryo growth. The promotions in embryo growth by increasing priming intensities for the GA-priming were very similar to those observed for the hydropriming. The GA-priming also did not simply mimic GA addition to the germination medium, which is known not to appreciably affect pre-germination embryo growth and germination speed of celery mericarps (Walker et al. 2021). We speculate that the GA-priming works with carrot, fennel, and parsley (this work, Pill and Kilian 2000; Attia et al. 2022) is due to GA-induced endosperm weakening (Walker et al. 2021; Steinbrecher and Leubner-Metzger 2017; Jacobsen et al. 1976). The negative effect of GA-priming on the two parsnip cultivars is more difficult to explain. Exogenous 100 µM GA_4+7_ did not have an appreciable effect on the germination of the two parsnip cultivars (Fig. 4), but high concentrations of exogenous GA_4+7_ (10–100 µM) are also known to inhibit coffee germination by causing cell death in the embryo (da Silva et al. 2005).

Additive priming with ABA (40–200 µM) increased germination speed and uniformity of species producing seeds with fully developed embryos such as brassicas, tomato, and cereals (Zhu et al. 2021; Yang et al. 2025; Srivastava et al. 2010; Yao et al. 2025a, 2025b; Finch-Savage and McQuistan 1991). The ABA-priming of these seeds resulted in increased abiotic stress tolerance of seedlings (drought, salinity), was manifested in boosting cuticular wax, cutin, and flavonoid biosynthesis in seedlings, and in some cases increased yield production. ABA-priming of sorghum seeds activated auxin biosynthesis in seedlings, enhancing auxin-mediated responses through ABA–IAA crosstalk and GA signalling (Yao et al. 2025b). Except that pre-treatment with 100 µM ABA synchronises carrot seed germination (Finch-Savage and McQuistan 1989). Nothing is published about the effects and mechanisms of ABA-priming for the Apiaceae crops. We found that ABA-priming with 100 µM ABA enhanced carrot and parsnip germination speed (GR_50%_) without appreciably affecting *G*_max_ (Fig. 5). The enhancement of carrot and parsnip germination speed for 14 days was associated with a similarly enhanced pre-germination embryo growth as for hydropriming. While 100 µM ABA added to the germination medium does not inhibit Apiaceae pre-germination embryo growth (this work, Homrichhausen et al. 2003; Walker et al. 2021), it inhibits carrot (Homrichhausen et al. 2003), celery (Walker et al. 2021), and parsnip (Fig. 1b) mericarp germination in a dose-dependent manner. The distinct effect of exogenous ABA and ABA-priming could be caused by enhanced ABA degradation during the priming which also leads to ~ 9.2-fold reduction in ABA contents in hydroprimed compared to untreated mericarps.

### Additive priming with ABA and gas plasma-activated water (GPAW) can enhance germination while retaining seed vigour

Gas plasma is often defined as ‘the fourth state of matter’ due to its high energetic state. In non-thermal (cold, non-equilibrium) atmospheric plasma, reactive species, including free radicals such as reactive oxygen species (ROS) and reactive nitrogen species (RNS), are produced at ambient temperature and atmospheric pressure (Bourke et al. 2018; Ito et al. 2018; Zhou et al. 2020). A technological application distinct from using it in the ‘gaseous’ form is to utilise plasma chemistry for the treatment of biological materials through ‘activating’ water. Gas plasma-activated water (GPAW) is produced by a gas plasma discharge at the gas–liquid interface and, in the case of air as the carrier gas, this initiates the formation of transient ROS and RNS such as hydroxyl (·OH) and nitric oxide (·NO) radicals, which react to form more stable compounds such as hydrogen peroxide (H_2_O_2_), nitrite (NO_2_^−^), and nitrate (NO_3_^−^). Treatment of mature seeds with a fully developed embryo with GPAW can release dormancy and enhance germination speed, as well as the growth of the emerged seedlings (Grainge et al. 2022a, 2022b; Ito et al. 2018; Bafoil et al. 2018; Ling et al. 2015). Published work with carrot (Guragain et al. 2025) and cumin (Shashikanthalu et al. 2020) demonstrates that plasma-treated MD mericarps are more wettable on their pericarp surfaces, have enhanced water uptake during imbibition, and their germination speed, as well as *G*_max_, are enhanced by the plasma treatment in the dry state, as well as by imbibition in GPAW. We demonstrate here (Fig. 9) that priming with GPAW enhanced carrot germination speed (*G*_50%_) beyond the enhancement achieved by hydropriming. Both GPAW-priming and hydropriming increased *G*_max_ and enhanced the salinity tolerance of mericarp germination (*G*_50%_ and *G*_max_). These beneficial effects of GPAW-priming were not caused by further enhancement of pre-germination embryo growth, which was roughly similar to hydroprimed carrot mericarps (Fig. 9d).

Seed vigour loss precedes seed viability loss in any storage/ageing environment, but the biochemical deterioration processes seem to differ quantitatively and qualitatively depending on the availability of oxygen, and the different temperature and humidity conditions (Bruggink et al. 1999; Corbineau 2024; Hay et al. 2022, 2019; Buitink and Leprince 2022; Zinsmeister et al. 2020). Different artificial ageing assays can be used to quantify seed vigour and viability loss during dry or wet storage/ageing conditions. Wet ageing assays correspond to seed storage conditions above 80% RH, whereas dry ageing assays correspond to RH below 75% (Zinsmeister et al. 2020) or 60% (Hay et al. 2022). Controlled deterioration (CD) tests at high moisture content and temperature within sealed aluminium foil packets (limited oxygen availability) were less suited as longevity assays to mimic dry storage/ageing environments (Hay et al. 2022). Gerna et al. (2022) provided evidence for different metabolic profiles in CD assays at dry (< 30%) versus wet (> 60%) conditions, and also concluded that seed bank storage is best simulated by dry CD conditions. On the other hand, CD tests at high moisture content (75% RH) and temperature (38 °C) and unlimited oxygen availability (air, norm pO_2_, not sealed) correlated best with “natural ageing” during long-term dry storage of Arabidopsis seeds (Renard et al. 2020), and proteomic analysis detected similar molecular mechanisms underpinning the Arabidopsis seed ageing in wet (CD at 85% RH, 40 °C) and dry (“natural”) ageing conditions (Rajjou et al. 2008). Recent work suggests that dry ageing assays with modifying oxygen or temperature are more suited to predict shelf-life (storability) under warehouse and genebank conditions (Hay et al. 2019, 2022; Buitink and Leprince 2022; Zinsmeister et al. 2020; Groot et al. 2012, 2025; Gerna et al. 2022). Further research into the similarities and differences of the molecular mechanisms underpinning dry and wet ageing assays at different temperatures and oxygen availability are therefore needed.

Reduced shelf-life (storability, longevity) due to faster deterioration processes during storage is a major drawback of primed seeds in many cases (Bradford 1986; Fabrissin et al. 2021; Pagano et al. 2023; Chojnowski et al. 1997; Argerich et al. 1989; Reed et al. 2022). Priming temperature and intensity, dry-back and storage conditions (oxygen, temperature and humidity) affect post-priming seed vigour and viability loss (Pagano et al. 2023; Hussain et al. 2015; Chiu et al. 2002; Owen and Pill 1994; Yan 2017; Schwember and Bradford 2011; Reed et al. 2022; Zinsmeister et al. 2020; Corbineau 2024; Groot et al. 2025). The impact of oxygen, temperature and humidity as the key factors affecting the longevity of primed seeds is still poorly investigated. Heat shock is often used to induce a longer shelf-life of primed seeds, but this constitutes an additional post-priming technological treatment step (Bruggink et al. 1999; Bruggink 2022; Batista et al. 2020; Khan et al. 2016; Fabrissin et al. 2021). Recent work with primed celery mericarps showed that for dry storage (< 43% RH) reduction of the oxygen level to 1% increased seed viability (shelf-life) by a factor of 11, while under wet storage conditions (60%) this oxygen effect was much less pronounced (Groot et al. 2025). In our work we did not analyse viability loss during storage, but used wet ageing assays to assess the vigour loss of unprimed and primed Apiaceae mericarps. Comparison of the carrot mericarp wet ageing sensitivity did not show a significant improvement by GPAW-priming compared to hydropriming (improvement factor 1.1, Supplementary Table S2). In contrast to carrot, the diaspore wet ageing sensitivity of non-Apiaceae species with a fully developed embryo was improved by GPAW-priming (Suppl. Fig. S4 and Tables S1, S2). When the wet ageing sensitivity of GPAW-primed and hydroprimed lettuce fruits (Suppl. Fig. S4) and tef grains (Fatelnig et al. 2024) were compared, their ageing resilience was increased ≥ threefold by the GPAW-priming. Wet ageing resilience of primed diaspores of other species, including cabbage, radish, swede and beetroot, was increased up to twofold by the GPAW-priming. GPAW-priming can therefore be used in many species to enhance germination while retaining diaspore ageing resilience.

We show here that ABA-priming of carrot and parsnip mericarps not only enhanced germination and prevented over-priming (Fig. 5), but it also increased their ageing resilience as quantified using the wet ageing assay (Figs. 6, 7, 8). Germination speed (GR_50%_) and *G*_max_ of carrot and parsnip mericarps primed for 14 days with 100 µM ABA were equal to or better compared to the non-primed control after the wet ageing assay (Fig. 8). The enhancement of carrot and parsnip germination speed for the 14-day ABA-priming was associated with a similarly enhanced pre-germination embryo growth as for hydropriming. While 100 µM ABA added to the germination medium inhibits Apiaceae mericarp germination in a dose-dependent manner, it does not inhibit Apiaceae pre-germination embryo growth (Fig. 1b this work, Homrichhausen et al. 2003; Walker et al. 2021; Walker et al. 2025). We speculate that ABA-priming acts by retaining programmes which are ABA-induced during seed maturation, including HSP and LEA proteins, which are required (Pagano et al. 2023; Fabrissin et al. 2021; Zinsmeister et al. 2020). ABA-insensitive pre-germination embryo growth as a unique feature of morphologically dormant Apiaceae seeds and fruits (this work, Homrichhausen et al. 2003; Walker et al. 2021, 2025) supports the use of ABA for these crop species. ABA-priming is therefore a promising technology to retain ageing resilience without the requirement of an additional post-priming treatment such as heat shock, especially for Apiaceae mericarps.

## Supplementary Information

Below is the link to the electronic supplementary material.

Supplementary file1 (PDF 1955 KB)

## Data Availability

The data generated in this study are available online in the electronic supplementary material and through figshare: 10.17637/rh.30095611.

## Abbreviations

ABA: Abscisic acid
CDT: Controlled deterioration treatment
CEL: Critical embryo length
CES: Critical embryo:seed length ratio
*cis*-OPDA: *cis*-(+)-12-Oxo-phytodienoic acid
H_2_O: Deionised water
E:S: Embryo:seed length ratio
GA: Gibberellin
*G*_max_: Maximal germination percentage
GPAW: Gas plasma-activated water
GR_50%_: Germination rate (speed) to 50%
IAA: Indole-3-acetic acid
MD: Morphological dormancy
PF: Puncture force
RH: Relative humidity
SMC: Seed moisture content
