# Correction: Enhancement of Apiaceae pre‑germination embryo growth, mericarp ageing resilience and germination differs between hormone, gas plasma, and hydropriming technologies

**DOI:** 10.1007/s00425-026-04966-4

**Published:** 2026-03-14

**Authors:** Lena M. M. Fatelnig, Matthew Walker, Giles Grainge, James E. Hourston, Sue Kennedy, Veronika Turečková, Ondřej Novák, Danuše Tarkowská, Miroslav Strnad, Kazumi Nakabayashi, Tina Steinbrecher, Gerhard Leubner‑Metzger

**Affiliations:** 1 Department of Biological Sciences, Seed Biology and Technology Group, Royal Holloway University of London, Egham, TW20 0EX UK; 2 Tozer Seeds Ltd, Cobham, KT11 3EH UK; 3 Syngenta Ltd., Jealott’s Hill International Research Centre, Bracknell, RG42 6EY UK; 4 Eden Research plc, Milton Park, Oxforshire, OX14 4SA UK; 5 Elsoms Seeds Ltd, Spalding, Lincolnshire, PE11 1QG UK; 6 Laboratory of Growth Regulators, Institute of Experimental Botany, Palacký University Olomouc, Czech Academy of Sciences and Faculty of Science, 77900 Olomouc, Czech Republic; 7 Department of Agro‑environmental Science, Obihiro University of Agriculture and Veterinary Medicine, Obihiro, Hokkaido 080‑8555 Japan

**Correction: Planta (2026) 263:35** 10.1007/s00425-025-04900-0

In this article, Jitka Široká was incorrectly added as the sixth author instead of Veronika Turečková.

The original article has been corrected.

