# Supplementary material for: Enhancement of Apiaceae pre-germination embryo growth, mericarp ageing resilience and germination differs between hormone, gas plasma, and hydropriming technologies
Source: Planta. 2026 Jan 3;263(2):35. doi: 10.1007/s00425-025-04900-0 (PMC12764683; doi:10.1007/s00425-025-04900-0)
Supplement: Supplementary file 1 — Supplementary file1 (PDF 1955 KB) [file 425_2025_4900_MOESM1_ESM.pdf]

Enhancement of Apiaceae pre-germination embryo growth, mericarp ageing tolerance and germination differs between hormone, gas plasma and hydropriming technologies

Lena M. M. Fatelnig[1], Matthew Walker[1,2], Giles Grainge[1,3], James E. Hourston[1,4], Sue Kennedy[5], Jitka Široká[6], Ondřej Novák[6], Danuše Tarkowská[6], Miroslav Strnad[6], Kazumi Nakabayashi[1,7,*], Tina Steinbrecher[1,*], Gerhard Leubner-Metzger[1,6,*]

[1] Department of Biological Sciences, Seed Biology and Technology Group, Royal Holloway University of London, Egham, TW20 0EX, United Kingdom, Web: 'The Seed Biology Place' - www.seedbiology.eu

[2] Tozer Seeds Ltd, Cobham, KT11 3EH, United Kingdom

[3] Syngenta Ltd., Jealott's Hill International Research Centre, Bracknell, RG42 6EY, United Kingdom

[4] Eden Research plc, Milton Park, Oxforshire, OX14 4SA, United Kingdom

[5] Elsoms Seeds Ltd, Spalding, Lincolnshire, PE11 1QG, United Kingdom

[6] Laboratory of Growth Regulators, Institute of Experimental Botany, Czech Academy of Sciences and Faculty of Science, Palacký University Olomouc, CZ-77900 Olomouc, Czech Republic

[7] Department of Agro-environmental Science, Obihiro University of Agriculture and Veterinary Medicine, Obihiro, Hokkaido, 080-8555, Japan

[*] For correspondence: E-mail Gerhard.Leubner@rhul.ac.uk or Tina.Steinbrecher@rhul.ac.uk or knakab@obihiro.ac.jp

Journal: Planta
Supplementary Information

Keywords: *Daucus carota* (carrot), *Pastinaca sativa* (parsnip), morphological seed dormancy, gas plasma seed priming, hormone seed priming, pre-germination embryo growth.

https://doi.org/10.1007/s00425-025-04900-0

© The Author(s) 2025. This is an open access article distributed under the terms of the Creative Commons Attribution License (https://creativecommons.org/licenses/by/4.0/) which permits unrestricted reuse, distribution, and reproduction in any medium, provided the original work is properly cited.

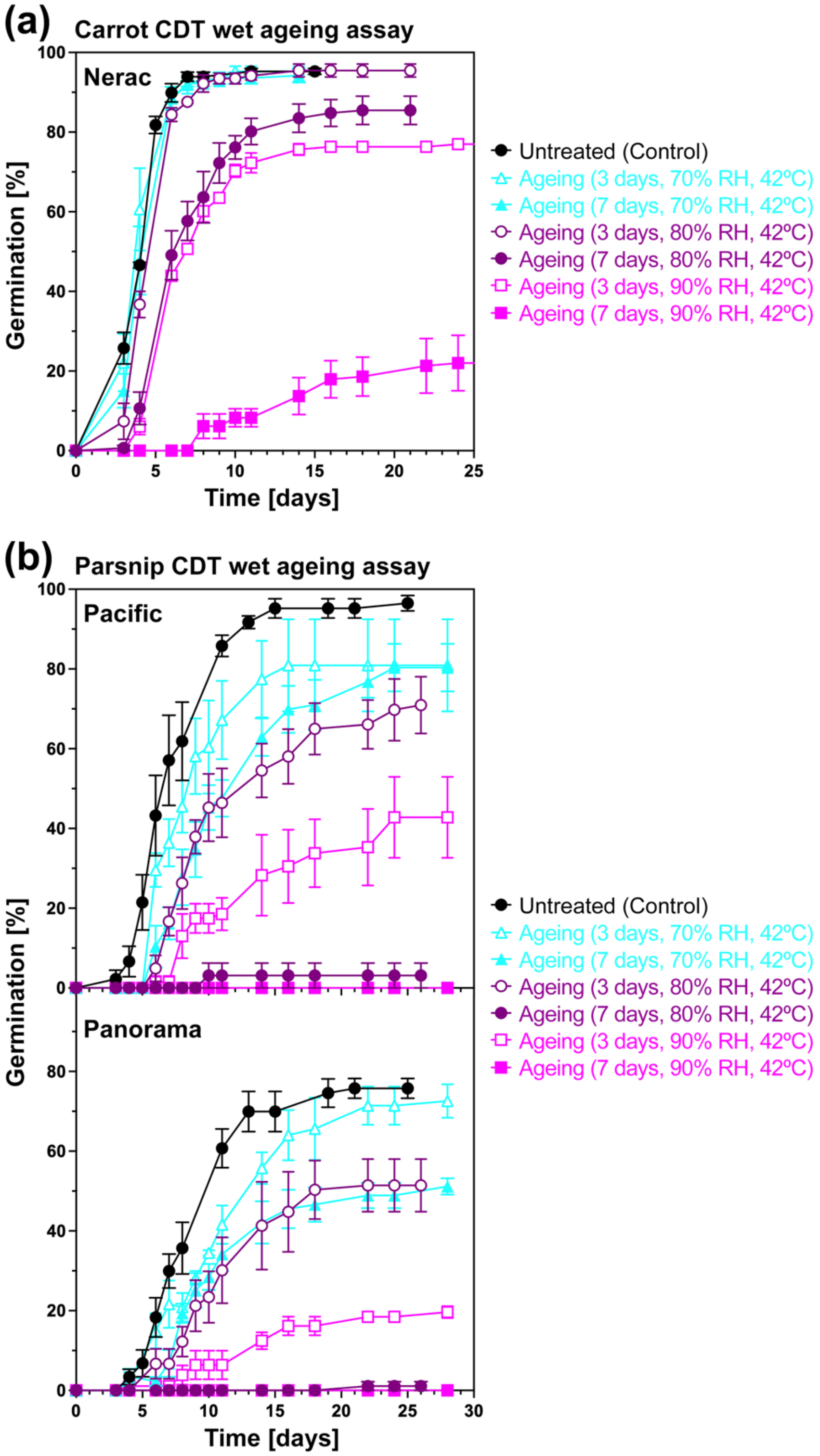

Supplementary Figure S1. Comparative analysis of the ageing sensitivities of untreated (control, unprimed) Apiaceae mericarps of using the wet ageing assay. **(a)** *Daucus carota* (carrot) cultivar Nerac. **(b)** *Pastinaca sativa* cultivars Pacific and Panorama. Germination analysis (imbibed at 20ºC in continuous white light) of mericarps was conducted after mericarps were subjected to the wet ageing assay, that is incubation at 42ºC at 70-90% relative humidity (RH) for 3 or 7 days. Mean ± SEM values of triplicate plates each with 50 (carrot) or 30 (parsnip) mericarps are presented.

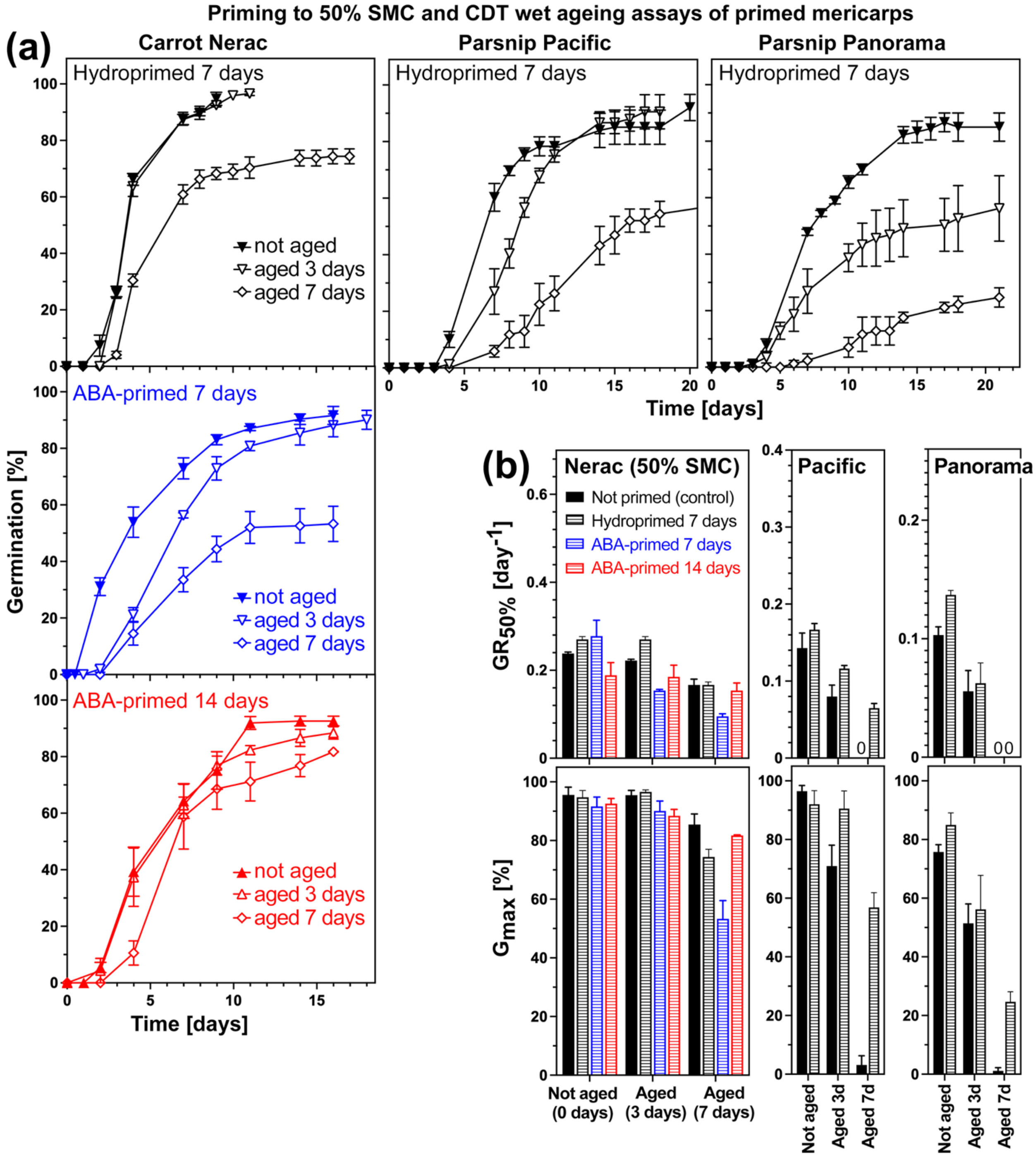

Supplementary Figure S2. Comparative analysis of the ageing sensitivities of untreated (unprimed, control), hydroprimed and ABA-primed *Daucus carota* (carrot) cultivar Nerac and *Pastinaca sativa* cultivars Pacific and Panorama using the wet ageing assay. **(a)** Germination analysis (imbibed at 20ºC in continuous white light) of mericarps was conducted after mericarps were first primed to 50% seed moisture content (SMC), and subsequently subjected to the wet ageing assay, that is incubation at 42ºC at 80% relative humidity (RH) for 3 or 7 days. Mean ± SEM values of triplicate plates each with 50 (carrot) or 30 (parsnip) mericarps are presented. **(b)** Quantified ageing sensitivity effects (wet ageing assay) of the mericarp germination rates $GR_{50\%}$, i.e. the inverse of the time required to reach 50% germination, and the maximal germination percentages (G_{max}); Mean ± SEM values derived from germination curves presented in panal (a).

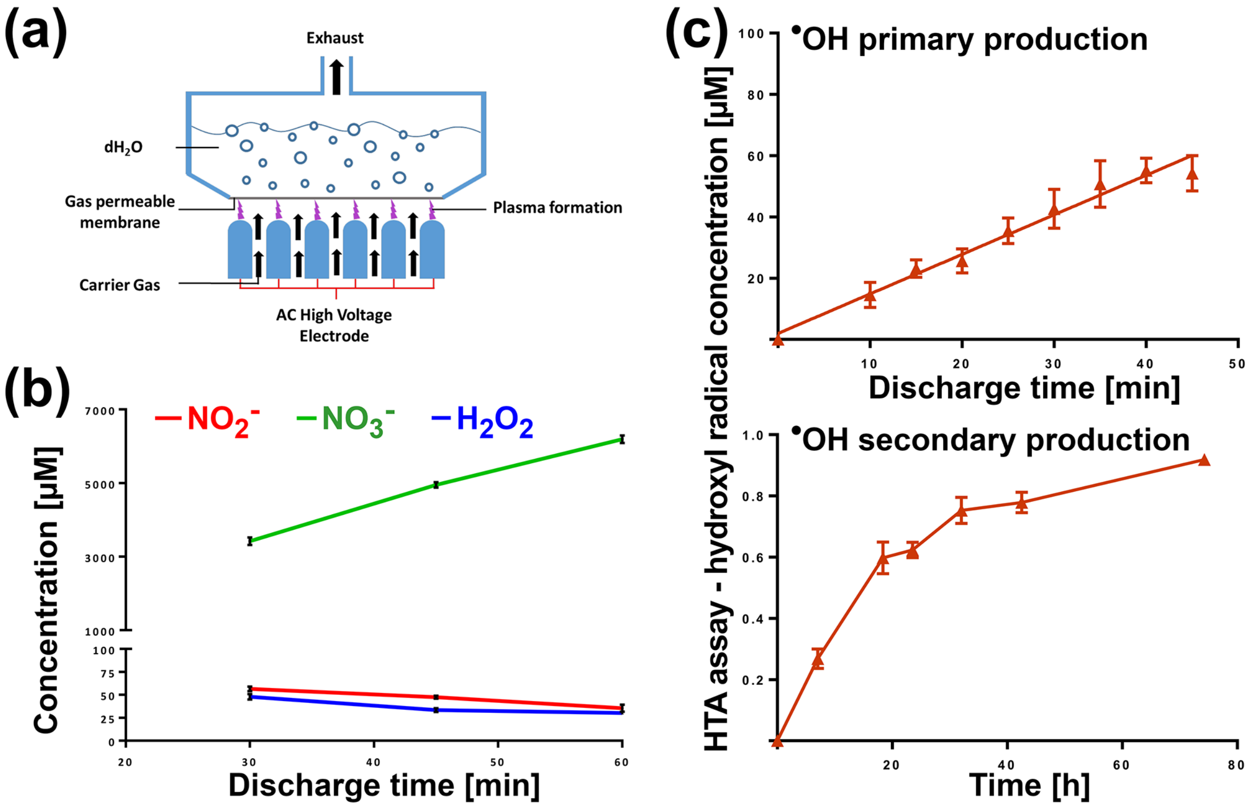

Supplementary Fig. S3 Diagram of the bubble reactor used to produce gas plasma activated water (GPAW) and chemical characterization of air-GPAW produced. **(a)** The bubble reactor includes 12 high voltage AC electrodes in a dielectric material fixed below a gas permeable stainless-steel membrane. Above the membrane is a tank containing 100 ml of deionised (dH_2O; water purifier system Select Purewater 300, Purite Ltd., Trevose, Pennsylvania, USA). Carrier gas flows past the electrode, and then through the membrane and dH_2O. For activation, plasma is formed between the electrodes and the membrane within the carrier gas and then flows through the membrane bubbling up through the water to produce the GPAW (from Grainge et al. 2022). **(b)** Quantified major chemical species produced with the bubble reactor included nitrite (NO_3^-), nitrate (NO_3^-) and hydrogen peroxide (H_2O_2) in the air-GPAW. **(c)** Primary and secondary production of hydroxyl radical ($^{\bullet}OH$) in the air-GPAW.

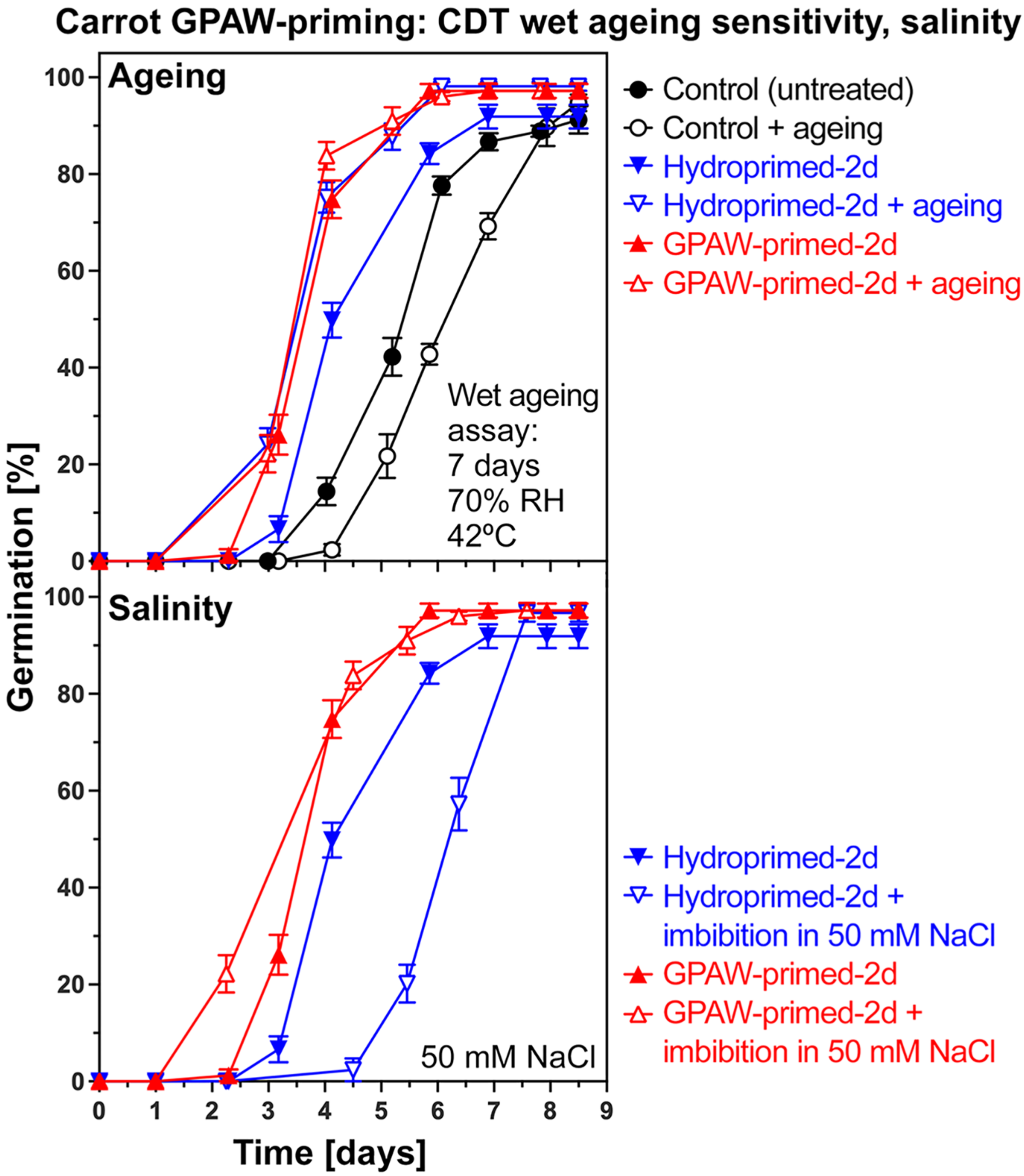

Supplementary Figure S4. Analysis of the ageing and salinity sensitivities of untreated (unprimed, control), hydroprimed and gas plasma-activated water (GPAW) primed *Daucus carota* (carrot) cultivar Newcastle using the wet ageing assay. Hydropriming or GPAW-priming were conducted for 2 days and a target seed moisture content of 70%. *Upper panel:* Germination analysis of mericarps after the wet ageing assay was conducted by incubation for 7 days at 42ºC at 70% relative humudity (RH). *Lower panel:* Germination analysis of hydroprimed or GPAW-primed mericarps in the presence of 50 mM NaCl (salinity). Mean ± SEM values of triplicate plates each with 50 mericarps are presented.

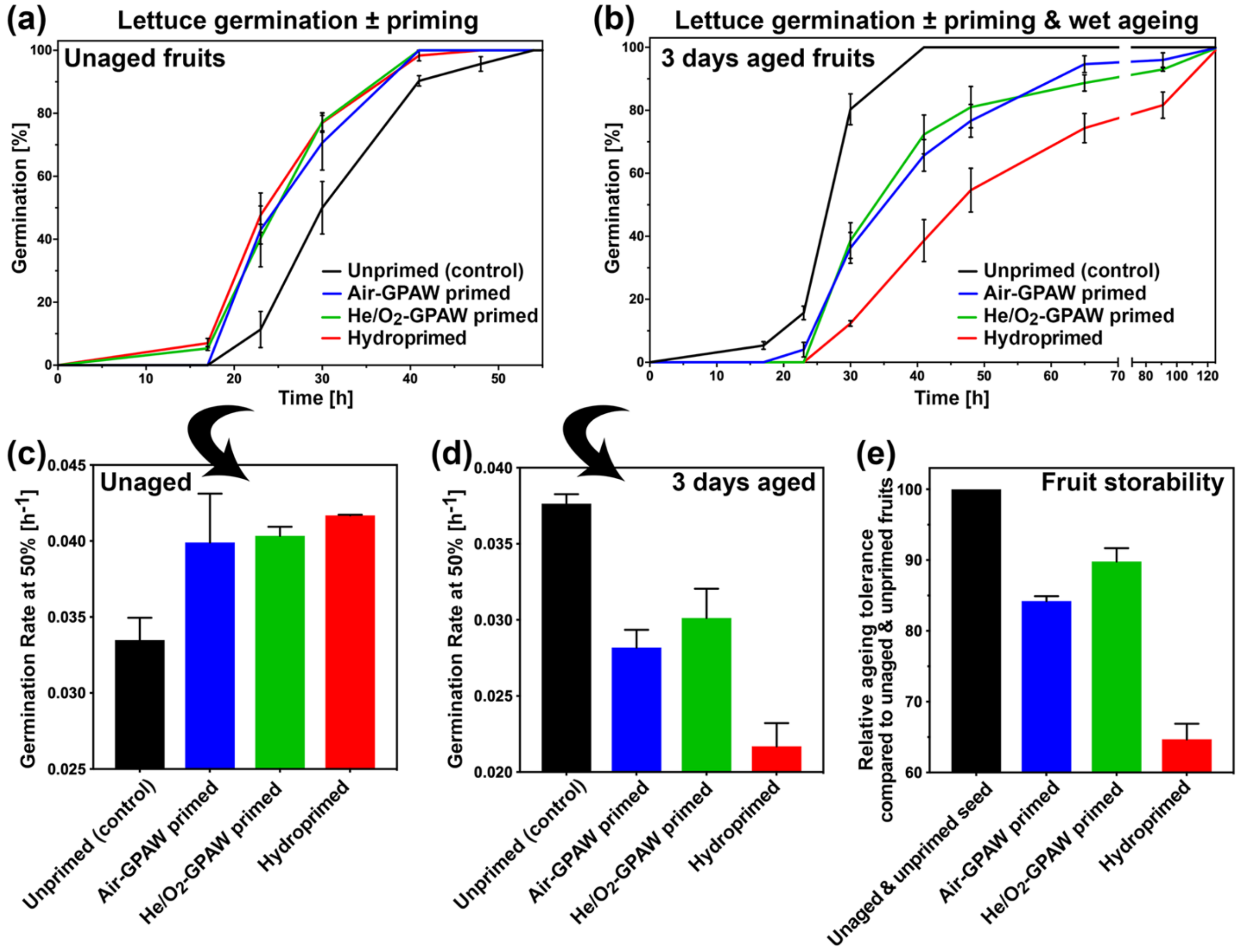

Supplementary Figure S5. Analysis of priming effects with gas plasma-activated water (GPAW) on germination on ageing resilience (storability) *Lactuca sativa* (lettuce) cultivar Grand Rapids fruits. The production of GPAW using either air (Air-GPAW) or He+O_2 admixture (He/O_2-GPAW) as carrier gas was as described in detail by Grainge *et al.* (2022). Hydropriming and GPAW-priming was with 60% target seed moisture content. **(a)** Germination analysis (imbibition at 24ºC in continuous white light) of unprimed (control), hydroprimed and GPAW-primed fruits. Mean ± SEM values of triplicate plates each with ~40 fruits are presented. **(b)** Germination analysis of unprimed and primed fruits after conducting the wet ageing assay, that is incubation of fruits at 50ºC, 70% relative humidity (RH) for 3 days, was used to estimate ageing sensitivity. **(c)** Germination rate (speed) $GR_{50\%}$ of not aged fruits, i.e. the inverse of the time required to reach 50% germination; Mean ± SEM values calculated from the germination curves in panel (a). **(d)** Germination rate (speed) $GR_{50\%}$ of fruits after wet ageing assay; Mean ± SEM values calculated from the germination curves in panel (b). **(e)** Relative ageing tolerance of treated fruits compared to the unprimed and unaged control. Mean ± SEM values.

Supplementary Table S1. Comparative germination results of seeds for the effects of gas plasma-activated water (GPAW) either "Not primed", "GPAW-primed" and "Hydroprimed" as compared to the seeds being subjected to the ageing assay by incubation at the conditions (temperature, relative humidity (RH), time) to quantify the ageing sensitivity and possible relative storability. Mean ± SEM values.

**Seed aging assay**		**Germination assay**			**Germination [%] Not primed**		**Germination [%] GPAW-primed**		**Germination [%] Hydroprimed**	
**Species**	**Common name**	**Aging conditions: Temp. [°C] / RH [%]**	**Aging time [days]**	**Imbibition time [days]**	**Mean**	**SEM**	**Mean**	**SEM**	**Mean**	**SEM**
Brassica oleraceae	Cabbage	Unaged control	0	4	72.2	1.2	77.0	4.7	82.5	2.4
		Aged: 40/60	7	4	45.5	4.1	75.8	3.2	55.9	2.5
Raphanus raphanistrum	Wild radish	Unaged control	0	5	44.4	8.2	81.3	11.9	76.1	6.7
		Aged: 40/60	7	5	30.4	6.8	82.0	10.9	60.2	9.2
Lactuca sativa	Lettuce	Unaged control	0	3	49.9	8.1	77.4	3.0	70.7	8.7
		Aged: 50/70	3	3	80.4	5.0	36.5	5.0	12.1	0.8
Brassica napobrassica	Swede	Unaged Control	0	2	11.1	3.9	75.2	2.2	36.0	4.0
		Aged: 42/70	7	2	11.9	6.2	66.0	1.5	41.9	3.8
Daucus carota	Carrot	Unaged Control	0	4	14.4	2.8	96.9	1.8	83.6	1.7
		Aged: 42/70	7	4	2.4	1.2	63.9	1.5	59.6	6.3
Beta vulgaris	Beetroot	Unaged Control	0	8	0	0	78.1	3.4	80.8	3.5
		Aged: 42/70	7	8	0	0	46.5	5.6	26.9	3.6

Supplementary Table S2. Comparative seed priming effect and seed storability (ageing resilience) for the effects of gas plasma-activated water (GPAW) on seeds either "GPAW-primed" or "Hydroprimed". Note that the column "Seed priming" provides a value for the priming effect of the GPAW-priming as compared to the hydropriming demonstrating that the GPAW-priming effect is either similar (values around 1) or better (Lettuce: value 3 means threefold as good). Note further that the column "Seed storability GPAW-primed/Hydroprimed" provides a value for the possible relative seed storability of the priming seed; values above 1 indicate potentially better storability of the GPAW-primed seeds.

**Species**	**Common name**	**Seed priming: GPAW-primed/Hydroprimed**[a]	**Seed storability: GPAW-primed**[b]	**Seed storability: Hydroprimed**[c]	**Seed storability: GPAW-primed/ Hydroprimed**
Brassica oleraceae	Cabbage	0.9	1.1	0.8	1.4
Raphanus raphanistrum	Wild radish	1.1	1.9	1.4	1.4
Lactuca sativa	Lettuce	1.1	0.7	0.2	3.0
Brassica napobrassica	Swede	2.1	6.0	3.8	1.6
Daucus carota	Carrot	1.2	4.5	4.1	1.1
Beta vulgaris	Beetroot	1.0	---	---	1.7
Eragrostis tef[d]	White tef	1.0	0.6	0.2	3.1
Eragrostis tef[d]	Brown tef	1.0	0.9	0.3	3.5

[a] Ratio between the unaged control germination percentages in Suppl. Table S1

[b] Ratio between the aged GPAW-primed and the unaged control germination percentages in Suppl. Table S1

[c] Ratio between the aged hydroprimed and the unaged control germination percentages in Suppl. Table S1

[d] Values from Figure 4 in Fatelnig et al. (2024)
